# No evidence for Fabaceae Gametophytic self-incompatibility being determined by Rosaceae, Solanaceae, and Plantaginaceae *S*-*RNase* lineage genes

**DOI:** 10.1186/s12870-015-0497-2

**Published:** 2015-06-02

**Authors:** Bruno Aguiar, Jorge Vieira, Ana E Cunha, Cristina P Vieira

**Affiliations:** Instituto de Investigação e Inovação em Saúde, Universidade do Porto, Rua Júlio Amaral de Carvalho 245, Porto, Portugal; Instituto de Biologia Molecular e Celular (IBMC), Universidade do Porto, Rua do Campo Alegre 823, Porto, 4150-180 Portugal

**Keywords:** Gametophytic self-incompatibility, Molecular evolution, *S-RNase* like genes, *Trifolium pratense*, *Medicago truncatula*, *Cicer arietinum*, *Cytisus striatus*

## Abstract

**Background:**

Fabaceae species are important in agronomy and livestock nourishment. They have a long breeding history, and most cultivars have lost self-incompatibility (SI), a genetic barrier to self-fertilization. Nevertheless, to improve legume crop breeding, crosses with wild SI relatives of the cultivated varieties are often performed. Therefore, it is fundamental to characterize Fabaceae SI system(s). We address the hypothesis of Fabaceae gametophytic (G)SI being *RNase* based, by recruiting the same *S-RNase* lineage gene of Rosaceae, Solanaceae or Plantaginaceae SI species.

**Results:**

We first identify *SSK1* like genes (described only in species having *RNase* based GSI), in the *Trifolium pratense*, *Medicago truncatula, Cicer arietinum, Glycine max*, and *Lupinus angustifolius* genomes. Then, we characterize the *S*-lineage *T2-RNase* genes in these genomes. In *T. pratense*, *M. truncatula,* and *C. arietinum* we identify *S-RNase* lineage genes that in phylogenetic analyses cluster with Pyrinae *S-RNases*. In *M. truncatula* and *C. arietinum* genomes, where large scaffolds are available, these sequences are surrounded by F-box genes that in phylogenetic analyses also cluster with *S-*pollen genes. In *T. pratense* the *S-RNase* lineage genes show, however, expression in tissues not involved in GSI. Moreover, levels of diversity are lower than those observed for other *S*-*RNase* genes. The *M. truncatula* and *C. arietinum S-RNase* and *S-*pollen like genes phylogenetically related to Pyrinae *S*-genes, are also expressed in tissues other than those involved in GSI. To address if other *T2-RNases* could be determining Fabaceae GSI, here we obtained a style with stigma transcriptome of *Cytisus striatus*, a species that shows significant difference on the percentage of pollen growth in self and cross-pollinations. Expression and polymorphism analyses of the *C. striatus S-RNase* like genes revealed that none of these genes, is the *S-*pistil gene.

**Conclusion:**

We find no evidence for Fabaceae GSI being determined by Rosaceae, Solanaceae, and Plantaginaceae *S-RNase* lineage genes. There is no evidence that *T2-RNase* lineage genes could be determining GSI in *C. striatus*. Therefore, to characterize the Fabaceae *S*-pistil gene(s), expression analyses, levels of diversity, and segregation analyses in controlled crosses are needed for those genes showing high expression levels in the tissues where GSI occurs.

**Electronic supplementary material:**

The online version of this article (doi:10.1186/s12870-015-0497-2) contains supplementary material, which is available to authorized users.

## Background

Useful agronomic traits can be found in wild populations of crop species. Nevertheless, a large fraction of species with hermaphroditic flowers have developed genetic mechanisms that allow the pistil to recognize and reject pollen from genetically related individuals (self-incompatibility; [1]), and this may affect the efficient incorporation of such traits into crop varieties. Self-incompatibility is, in general, evolutionarily advantageous, because it promotes cross-fertilization, and thus inbreeding depression avoidance.

Fabaceae is an economically important plant family with a large number of self-incompatible species (62.3% in Caesalpinioideae, 66.7% in Mimosoideae, and 22.1% in Papilionoideae sub families; [2]), that have been reported often as showing self-incompatibility of the gametophytic type (GSI; [1-9]). In GSI, if the specificity of the haploid pollen grain matches either one of the diploid pistil, an incompatible reaction occurs, leading to the degradation of the pollen tube within the pistil [10]. It should be noted, however, that in all Fabaceae species where pollen tube growth was assessed in controlled crosses, only in species of the genus *Trifolium* the GSI reaction seems to be complete and takes place in the stlyle [3,11] as observed in Rosaceae (Rosidae; for a review see [12,13]), Solanaceae (Asteridae; [14]) and Plantaginaceae (Asteridae; [15,16]) SI species. In other species such as *Vicia faba* [17], *Lotus corniculatus* [18], *Cytisus striatus* [7], *Coronilla emerus* and *Colutea arborescens* [19] there is, however a significant difference on the percentage of pollen growth in self and cross-pollinations. In *C. striatus*, one of the species here studied, the percentage of ovules that are penetrated by pollen tubes is 72% in hand self-pollinated flowers compared with the 90.6% when hand cross-pollinations are performed [7]. These authors have shown that an important fraction of self pollen grains collapse along the style, as observed in Rosaceae, Solanaceae and Plantaginaceae SI species.

Although the molecular characterization of the Fabaceae *S*-locus has never been performed, some authors have suggested that in Fabaceae GSI is *RNase* based [1,2,4-9]. Nevertheless, there are other GSI systems, such as that present in Papaveraceae [for a review see [20]]. Moreover, late-acting SI (LSI), so called because rejection of self-pollen takes place either in the ovary prior to fertilization, or in the first divisions of the zygote [21], has been described in Fabaceae [18,22-24]. It should be noted that, LSI can also be of the gametophytic type [21]. In Fabaceae, however, the genetic basis of the different mechanisms that control LSI are mostly unknown, and thus, in this work we only address the possibility that Fabaceae GSI is determined by a *S-RNase* gene that clusters with those of the well characterized Rosaceae [12,13], Solanaceae [14] and Plantaginaceae [15,16] species. The most common ancestor of Fabaceae (Rosidae) and Rosaceae species lived about 89–91 million years ago (MYA; [25]). Since, according to phylogenetic analyses of the *T2-RNases*, *RNase* based GSI has evolved only once, before the split of the Asteridae and Rosidae, about 120 MYA [26-28], at least some Fabaceae SI species are expected to have this system. Therefore, in principle, a homology based approach could be used to identify the putative pistil *S*-gene in Fabaceae species.

Three amino acid patterns (amino acid patterns 1 and 2 that are exclusively found in proteins encoded by *S-RNase* lineage genes, and amino acid pattern 4 that is not found in any of the proteins encoded by *S-RNase* lineage genes), allow the distinction of *S-RNase* lineage genes from other *T2 -RNase* genes [28,29]. These patterns can be used to easily identify putative *S*-lineage genes using blast searches. The results can be further refined by selecting only those genes that encode basic proteins (isoelectric point higher than 7.5) since S-RNases have an isoelectric point between 8 and 10 [30]. Furthermore, the number of introns can also be used to select *S*-lineage genes since *S-RNases* have one or two introns only (Figure one in [16]). Phylogenetic analyses where a set of reference genes are used, can then be performed to show that such genes belong, indeed, to the *S*-lineage. Nevertheless, in order to show that the identified genes are the pistil *S*-gene, it is necessary to show that they are highly expressed in pistils, although they can show lower expression in stigma and styles (see references in [31]). In *Malus fusca* where a large number of transcriptomes (flowers, pedicel, petal, stigma, style, ovary, stamen, filaments, anthers pollen, fruit, embryo and seed) have been analysed the same pattern is observed (CP Vieira, personal communication). Moreover, it is necessary to show that they have high polymorphism levels, that there is evidence for positive selection, and that in controlled crosses they co-segregate with *S-*locus alleles (see references in [31]).

The pollen component(s), always an F-box protein, has been identified as one gene in *Prunus* (Rosaceae; the gene is called *SFB* [32-37]), but multiple genes in Pyrinae (Rosaceae; the genes are called *SFBB*s [38-45]) and Solanaceae (called *SLFs*; [46-48]). F-box genes belong to a large gene family, and so far, no typical amino acid patterns have been reported for *S*-locus F-box protein sequences. Therefore, in non-characterized species, it is difficult to identify the pollen *S*-gene(s) using sequence data alone. In contrast to the *S-RNase* gene, Pyrinae *SFBB* genes show low polymorphism and high divergence [41-45]. Pollen *S*-gene(s) is (are), however, expected to be mainly expressed in the pollen [32,33,40,46,47].

Although the mechanism of self pollen tubes recognition is different when one or multiple *S*-pollen genes are involved [35,49], SSK1 (SKP1 like) proteins are involved in the self-incompatibility reaction in Rosaceae, Solanaceae and Plantaginaceae species, where GSI systems are well characterized. SKP1 like proteins are adapters that connect diverse F-box proteins to the SCF complex, and that are necessary in a wide range of cellular processes involving proteosome degradation (see references in [50]). SSK1 proteins have been described only in species having *RNase* based GSI [50-53], and thus, their presence has been suggested as a marker for *RNase* based GSI [53]. These proteins are highly conserved and have a unique C-terminus, composed of a 5–9 amino acid residues following the conventional “WAFE” motif that is found in most plant SKP1 proteins [52]. Therefore, the genes encoding such proteins can be easily retrieved using blast searches. In Solanaceae, Plantaginaceae, and Pyrinae, SSK1 proteins are expressed in pollen only [50-53], but in *Prunus* they are also expressed in styles [54].

To identify *T2-RNases* that could be *S*-locus candidate genes in Fabaceae subfamily Papilionoideae, in this work, we characterized the *S*-lineage *T2-RNase* genes in five genomes of species belonging to three major subclades: *Trifolium pratense, Medicago truncatula*, and *Cicer arietinum* from the inverted-repeat-lacking clade (IRLC), *Glycine max* from the millettioid clade, and *Lupinus angustifolius* from the genistoid clade. *Trifolium* and *Medicago* are the most closely related genera, and they share the most recent common ancestor, about 24 MYA [55]. *Cicer* is diverging from these two genera for about 27 MY. *Glycine* is diverging from species of the IRLC clade for about 54 MY, and *Lupinus* is diverging from these for about 56 MY [55]. Except for *T. pratense*, all these species are self-compatible. Nevertheless, the *S*-locus region could, in principle, be present, although the *S*-locus genes are expected to be non-functional [56]. Compatible with this view, sequences closely related to the *SSK1* genes are here identified in *T. pratense, M. truncatula, C. arietinum*, and *G. max* genomes. In *T. pratense, M. truncatula* and *C. arietinum* we identify *S-RNase* lineage genes that in phylogenetic analyses cluster with Pyrinae *S-RNases*. Furthermore, in *M. truncatula* and *C. arietinum* genomes, where large scaffolds are available, these sequences are surrounded by F-box genes that in phylogenetic analyses cluster with *S*-pollen genes. Nevertheless, none of these genes show expression only in tissues related with GSI. Moreover, *T. pratense* genes present levels of diversity lower than those of the characterized *S-RNase* genes. We also obtained a style with stigma transcriptome for *Cytisus striatus*, a species where self-pollen grains have been reported to collapse along the style, although partially [7]. Once again, we found two genes that encode proteins showing the typical features of *SSK1* genes and three *T2-RNase* like sequences, but none of these genes shows expression and variability levels compatible with being the *S-RNase* gene. Thus, we find no evidence for *RNase* based GSI in *C. striatus*. The data here presented supports the hypothesis that Fabaceae GSI is not determined by Rosaceae, Solanaceae, and Plantaginaceae *S-RNase* lineage genes. Alternative hypotheses are here discussed regarding the presence of *SSK1* genes and Fabaceae GSI system.

## Results

### ***SSK1*** like genes in Fabaceae

*SSK1* genes(s) are restricted to species having *RNase* based GSI [50-53]. The presence/absence of this gene(s) has been reported as a diagnosis marker for the presence/absence of *RNase* based GSI [50-53]. The protein encoded by SSK1 has an unique C-terminus, composed of 5–9 amino acid residues, following the conventional “WAFE” motif [52]. In Rosaceae, this amino acid tail shows the conserved sequence “GVDED” (Additional file 5 in [54]). In Solanaceae and Plantaginaceae this motif is not so well conserved but a D residue is always found at the last position of the motif. It should be noted that most of the Fabaceae genomes that are available are from self-compatible species, and thus, *SSK1* genes may be non-functional, or not involved in SI pathway. Therefore, when retrieving the sequences we allowed for some variability regarding these motifs (see Methods).

When using these features and the NCBI flowering plant species database, we retrieved 21 sequences from Solanaceae (three), Plantaginaceae (one), Rosaceae (eight), Fabaceae (five), Malvaceae (one), Rutaceae (one), Euphorbiaceae (one) and Salicaceae (one) species. Two other sequences, *cy54873-cy21397* (this gene is the result of merging two sequences - *cy54873g1* and *cy21397g1* that overlap in a 22 bp region at the end of one and beginning of the other; PRJNA279853; http://evolution.ibmc.up.pt/node/77; http://dx.doi.org/10.5061/dryad.71rn0) and cy41479g1 (PRJNA279853; http://evolution.ibmc.up.pt/node/77; http://dx.doi.org/10.5061/dryad.71rn0) were identified in the *C. striatus* style with stigma transcriptome. These *C. striatus* sequences are incomplete at the 5′ region, since using blastx, the first 77 amino acids of SSK1 proteins are not present in these sequences. On the other hand, these sequences are complete at the 3′ region since their putative amino acid sequence presents the Rosaceae GVDED motif after the WAFE motif.

**Figure 1:**
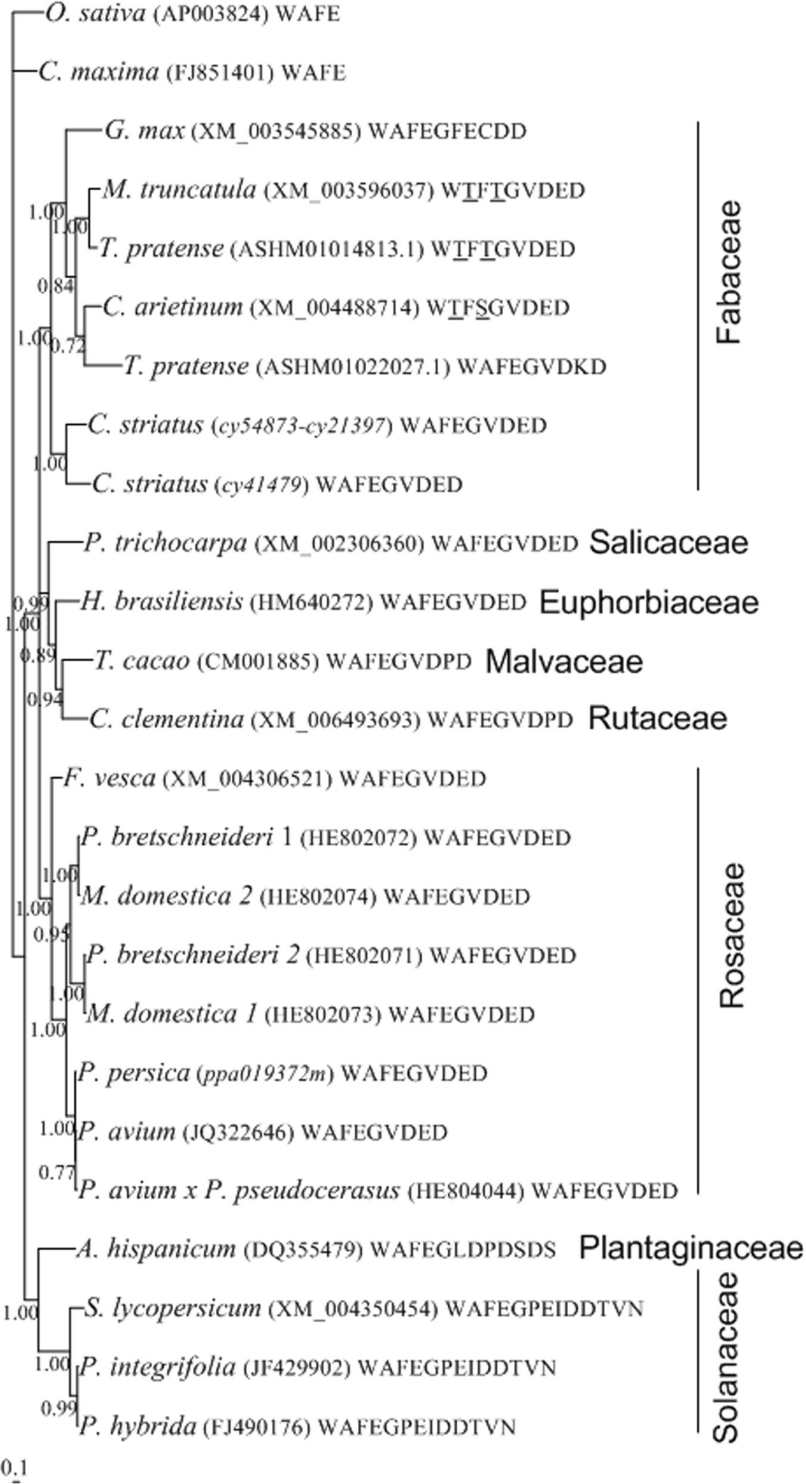
Bayesian phylogenetic tree showing the relationship of *SSK1* like genes in flowering plants presenting these genes, available at GenBank (sequences were aligned using the Muscle algorithm). Numbers below the branches represent posterior credibility values above 60. The tree was rooted using *Oryza sativa* [GenBank:AP003824] and *Citrus maxima* [GenBank:FJ851401] genes that encode proteins not presenting the C-terminus amino acid motif following the conventional “WAFE” motif. The C-terminus amino acid motif following the conventional “WAFE” of the proteins encoded by each *SSK1* gene is also presented. Amino acids that are different from the “WAFE” motif are underlined.

The phylogenetic relationship of the 23 *SSK1* sequences, as well as the C-terminus sequence motif of the proteins they encode is presented in Figure 1 (see also Additional file 1). Fabaceae *SSK1* like genes are more closely related to Rosaceae *SSK1* sequences than to those from Solanaceae and Plantaginaceae (Figure 1), according to the known relationship of the plant families. It should be noted that only the two *C. striatus* deduced proteins present the Rosaceae GVDED motif after the WAFE motif. The *T. pratense ASHM01022027.1*, and *G. max XM_003545885* genes encode proteins that present the WAFExxxxD motif, described for Solanaceae and Plantaginaceae SSK1. The presence of *SSK1* genes in Fabaceae is, thus, consistent with the claims of *RNase* based GSI in Fabaceae.

SSK1 proteins showing the Rosaceae motif are also found in *Hevea brasiliensis* (Euphorbiaceae) and *Populus trigonocarpa* (Salicaceae). None of these species, or species of these families, has been described as having GSI. Furthermore, in *Citrus clementina* SSK1 like proteins present a proline instead of a glutamic acid in the Rosaceae WAFEGVDED motif. *Citrus* species present GSI and cytological analysis showed that growth of pollen tubes is arrested in different regions depending on the species analysed [57]. In *C. clementina* pollen tubes are arrested in the upper styles [58]. *RNase* activity has been identified in stigmas and pistils of *C. reticulata* [59,60] and also in ovaries of *C. grandis* [61], but the genetic mechanism is not clear yet [62]. Indeed, in the comparative transcriptome analyses of stylar cells of a self-incompatible and a self-compatible cultivar of *C. clementina*, no *T2-RNases* where identified [63], rising doubts if GSI is *RNase* based in *C. clementina*. In *T. cacao* (Malvaceae) a SSK1 like protein with the same pattern as in *C. clementina* has also been identified. In this species self-pollen tubes grow to the ovary without inhibition, and self-incompatibility occurs at the embryo sac [64], and not in the style. Nevertheless, other Malvaceae species such as diploid species of the *Tarasa* genera present GSI (Table 1 in [65]), although the genetic mechanism is unknown.

**Table 1:** ***M. truncatula*** **,** ***C. arietinum*** **,** ***G. max*** **,** ***L. angustifolius T2-RNases*** **larger than 500 bp, that encode putative proteins not presenting in their amino acid sequence amino acid pattern 4 according to Vieira,** ***et al.*** [28]

**Locus**	**Gene code**	**IP**	**Intron number**	**Motif 1**	**Motif 2**	**Motif 4**	**Location**
*T. pratense*							
[GenBank*:ASHM01010303*] {	*Tp1*	9.20	1	FVIHGLWPS*R*	WP*S*LKYN	-	ASHM01010303: 956… 1742
[GenBank*:ASHM01021082*]	*Tp2*	8.82	1	FTIHG*M*WPSN	WP*SY*TSP	-	ASHM01021082: 467… 1277
[GenBank*:ASHM01011821*]	*Tp3*	7.57	1	FSVHG*V*WP*T*N	WPDLKGG	-	ASHM01011821: 2194… 2920
[GenBank*:ASHM01032414*]	*Tp4*	9.20	1	F*V*IHGLWP*VF*	WP*S*LKYN	-	ASHM01032414: 1330… 2116
[GenBank*:ASHM01032369* ]+	*Tp5*	9.92	1	FTIHGLWPSN	WPNLKWT	-	ASHM01032369: 1121… 2019
[GenBank*:ASHM01005450*]	*Tp6*	9.18	1	FS*L*HGLWPSN	WP*S*LFVG	-	ASHM01005450: 3673… 4373
[GenBank*:ASHM01035891*]	*Tp7*	9.06	1	FTIHGLWPSN	WPNLL*M*V	-	ASHM01035891: 1083… 2003
[GenBank*:ASHM01035915*]	*Tp8*	9.51	1	FTLHGIWPSN	WPDLKGQ	-	*ASHM01035915*: 1152… 2109
[GenBank*:ASHM01087496*]	*Tp9*	8.11	1	FSIHGLWP*Q*N	WP*S*LTGN	-	*ASHM01087496*: 1… 681
[GenBank*:ASHM01016923*]	*Tp10*	6.87	1	FSIHGLWPQN	WPSLTGK	-	*ASHM01016923*: 1540… 2300
[GenBank*:ASHM01047800*]	*Tp11*	9.48	1	FTTHGLWPSN	WPNLKGP	-	*ASHM01047800*:1… 629
[GenBank*:ASHM01027928*]	*Tp12*	8.75	1	FTIHGLWPSN	WPNLLSN	-	*ASHM01027928*:226… 1002
[GenBank*:ASHM01008805*]	*Tp13*	8.85	1	FSIHGLWP*Q*N	WP*S*LTGN	-	*ASHM01008805*:250… 977
[GenBank*:ASHM01049573*]	*Tp14*	8.64	1	FTTHGLWPSN	WPNLKGP	-	*ASHM01049573*:1… 575
[GenBank*:ASHM01036061*] {	*Tp15*	9.20	1	F*V*IHGLWPSI	WP*S*LKYN	-	*ASHM01036061*: 956… 1742
*M. truncatula*							
[GenBank:AC123571.8 (*Medtr5g022810*)]	*Mt1*	7.57	2	F*VM*HGLWPAN	WPDLLVY	-	Mt5:8,780,338..8,781,194
[GenBank:AC149207.1 (*Medtr2g021830*)]	*Mt2*	7.06	1	FT*L*HGLWPSN	WPNLFGA	-	Mt2:7,405,970..7,406,697
[GenBank:AC149207.2]	*Mt3*	8.57	2	FTVHGLWPSN	WP*S*VTTT	-	Mt2:7,383,161..7,384,370
[GenBank:AC149269.11 (*Medtr6g090200*)]	*Mt4*	6.39	1	FTI*Q*GL*F*PNN	WINYIGD	-	Mt6:22,040,215..22,039,455
[GenBank:AC159124.1] >	*Mt5*	9.06	2	*L*TVHGLWPSN	WPDVGGT	-	Mt2:7,374,496..7,375,004
[GenBank:AC196855-3 (*Medtr2g104330*)] {	*Mt8*	8.05	1	FT*L*HG*F*WPSN	*Y*P*FD*FNT	*DFN*TTK	Mt2:34,011,354..34,010,761
[GenBank:CR936945 (*Medtr5g086410*)]	*Mt9*	8.45	1	*L*TIRGLWPST	WP*S*LNSG	-	Mt5:36,330,498..36,331,243
[GenBank:CU459033 (*Medtr5g086770*)]	*Mt10*	5.78	2	F*K*I*W*GLWP*VR*	WP*S*LFGP	*SLF*GPD	Mt5:36,498,402..36,499,282
[GenBank:CT573354]	*Mt12*	8.83	1	FTIHGVWPSN	WPRLDTA	-	Mt3:9,158,726..9,157,789
[GenBank:CU026495]	*Mt13*	8.83	1	FTIHGLWPSN	WPRLDTA	-	Mt3:9,139,338..9,138,417
[GenBank:AC126012 (*Medtr5g0977101*)]	*Mt14*	5.20	1	F*LLY*G*A*WP*V*D	W*R*D*I*KNG	*IKN*GDD	Mt5:41,755,316..41,755,711
[GenBank:AC233685_48.1 (XM003637773)]	*Mt16*	9.21	1	FTIHGLWP*T*N	WPDVIHG	-	MtU:12,302,642..12,303,437
[GenBank:*Medtr2g021910.*2]	*Mt17*	8.54	2	*L*TIHGLWPSN	WP*SI*YGD	*IYG*DDD	Mt2:7,440,534..7,441,199
*Mettr2g021910.2*	*Mt18*	8.40	2	*L*TIHGLWPSN	WP*TI*YGS	*IYG*SDD	Mt2:7,445,004..7,445,674
[GenBank:AC124218 (XM003624084)]	*Mt20*	8.82	1	FTIHGLW*V*EN	WP*S*LYQK	*LY*QKSS	Mt7:22,479,456..22,480,238
[GenBank:CM001222 {	*Mt23*	9.55	1	FSIHGLWP*T*N	WPDAVYG	-	Mt6:12,596,544..12,596,922
BT148419]	*Mt24*	5.77	1	FTIHGLWPD*Y*	WP*S*LS*C*G	-	MtT:10,244,733..10,244,880
[GenBank:BT136026 (AFK35821)]	*Mt25*	6.86	3	FTFILQWPGS	WP*S*LR*C*P	CPR*L*NN	Mt5:17,636,584..17,636,691
[GenBank:AW776643] >	*Mt26*	8.47	n.a	F*G*IHGLWP*T*N	WPNLLE*W*	-	-
*C. arietinum*							
[GenBank:XP_004503396 (NC021165)]	*Ca1*	8.62	1	FTIHGLWPSN	WPNLKGQ	-	Ca6:2,486,865..2,487,892
[GenBank:CM001766.1]	*Ca2*	9.35	1	*L*TVH*I*LW*GT*N	W*N*D*H*SFC	-	Ca3:9,734,288..9,735,009
[GenBank:XP_004486305 (CM001764.1)] {	*Ca3*	9.44	1	FTVHGLWPSN	WPNLFGN	-	Ca1:34,647,053..34,647,728
[GenBank:XP_004486305 (CM001764.1)]	*Ca4*	7.59	1	FTVHGLWPSN	WPNLFGN	-	Ca1:5,252,166..5,252,821
[GenBank:CM001767.4]	*Ca5*	8.47	1	FTIHGLWP*Y*N	WPDLKGQ	-	Ca4:42,156,653..42,157,571
[GenBank:CM001767.3] {	*Ca6*	6.43	2	F*I*IHGLWPSN	WPNLKGQ	-	Ca4:3,880,028..3,880,839
[GenBank:CM001768.1] {	*Ca7*	9.02	1	FTIHGLWPSN	W*S*NLKGQ	-	Ca5:12,546,395..12,547,197
[GenBank:CM001768.2]	*Ca8*	9.24	1	FTIHGLWP*F*N	WPNL*N*GQ	-	Ca5:11,753,113..11,753,945
[GenBank:CM001769.1]	*Ca9*	9.28	1	FTIHGLWPNN	WP*S*LIKG	-	Ca6:45,431,765..45,432,712
[GenBank:XP_004505385 (CM001769.2)]	*Ca10*	8.85	1	FTIHGLWPSN	WPNLKGQ	-	Ca6:16,977,346..16,978,256
[GenBank:CM001769.4]	*Ca11*	8.61	1	FTLHGLWPSN	WPNL*N*GV	-	Ca6:31,097,283..31,098,019
[GenBank:CM001769.5] {	*Ca12*	7.79	1	FTIHGLWPSN	WP*S*LT*M*S	-	Ca6:28,777,475..28,778,149
[GenBank:CM001769.6] {	*Ca13*	9.03	1	FTLHGLWPSN	WPNL*N*GG	-	Ca6:33,284,148..33,284,935
[GenBank:CM001769.7] >	*Ca14*	8.27	1	*KI*IHGLWPSN	*PSLT*KSQ	-	Ca6:28,744,494..28,745,117
[GenBank:CM001769.8]	*Ca15*	8.80	1	FTIHGLWPSN	WPNLKGQ	-	Ca6:2,486,787..2,487,895
[GenBank:XP_004507007 (CM001769.9)]	*Ca16*	9.17	1	FTIHGLW*GTN*	WPDVINQ	-	Ca6:52,088,714..52,089,462
[GenBank:XP_004503396 (CM001769.10)]	*Ca17*	9.09	1	FTIHGLWPSN	WPNLKGQ	-	Ca6:2,486,751..2,487,895
[GenBank:XP_004505385 (CM001769.11)]	*Ca18*	8.85	1	FTIHGLWPSN	WPNLKGQ	-	Ca6:16,977,346..16,978,256
[GenBank:XP_004514375 (gi484567706)] >	*Ca19*	8.47	1	FTLHGLWPSN	WPNL*N*GV	-	scaffold485:91,749..192,162
[GenBank:XP_004506021 (gi484571392)]	*Ca20*	9.02	1	F*K*IHGLWPSN	WP*S*LIDS	-	Ca6:28,325,256..28,326,148
[GenBank:XP_004515186 (gi484566269)]	*Ca21*	9.16	1	F*K*IHGLWPNT	WP*S*LKKS	-	scaffold948:113,466..114,365
*G. max*							
[GenBank:CM000836]	*Gm1*	9.05	1	FTIHGLWP*Q*N	WPNL*N*TQ	-	GM03: 42522935… 42523824
[GenBank:XP_003548020)] {	*Gm2*	5.71	2	FTI*SYFRPR*K	WPDLTTD	-	GM16: 30294108… 30295346
[GenBank:NP_001235172]	*Gm3*	6.80	2	FTI*SY*L*H*P*MR*	WPDLRTD	-	GM02: 5707162… 5708520
[GenBank:XP_003519927]	*Gm4*	5.47	2	FTI*SYFR*P*R*K	WPDLRTD	-	GM02: 5686955… 5688178
[GenBank:XP0035181161]	*Gm5*	7.49	2	FTI*SY*L*H*P*MR*	WPDLRTD	-	GM02: 5682344… 5683625
[GenBank:XP003518119]	*Gm6*	6.30	2	FTI*SY*L*H*P*MR*	WPDLRTD	-	GM02: 5698841… 5700244
[GenBank:CM000853]	*Gm7*	8.61	3	FSIHGLWPN*F*	WA*S*LS*C*A	-	GM20:5212321… 5214271
*L. angustifolius*							
[GenBank:AOCW01152977]	*La1*	9.04	0	FTLHGLWP*I*N	WPNL*N*GK	-	scaffold92513_2

IP- isoelectric point.

Underscored are amino acids that are not allowed in the motifs of [28].

+ sequences presenting stop codons in the putative coding region.

{ sequences where gaps were introduced to avoid stop codons in the putative coding region.

> very divergent sequences that, although they present all the criteria of *S*-lineage *S-RNase* genes, were not included in phylogenetic analyses.

### ***T. pratense, M. truncatula, C. arietinum, G. max*** and ***L. angustifólioT2-RNaseS***-lineage genes

**Figure 2:**
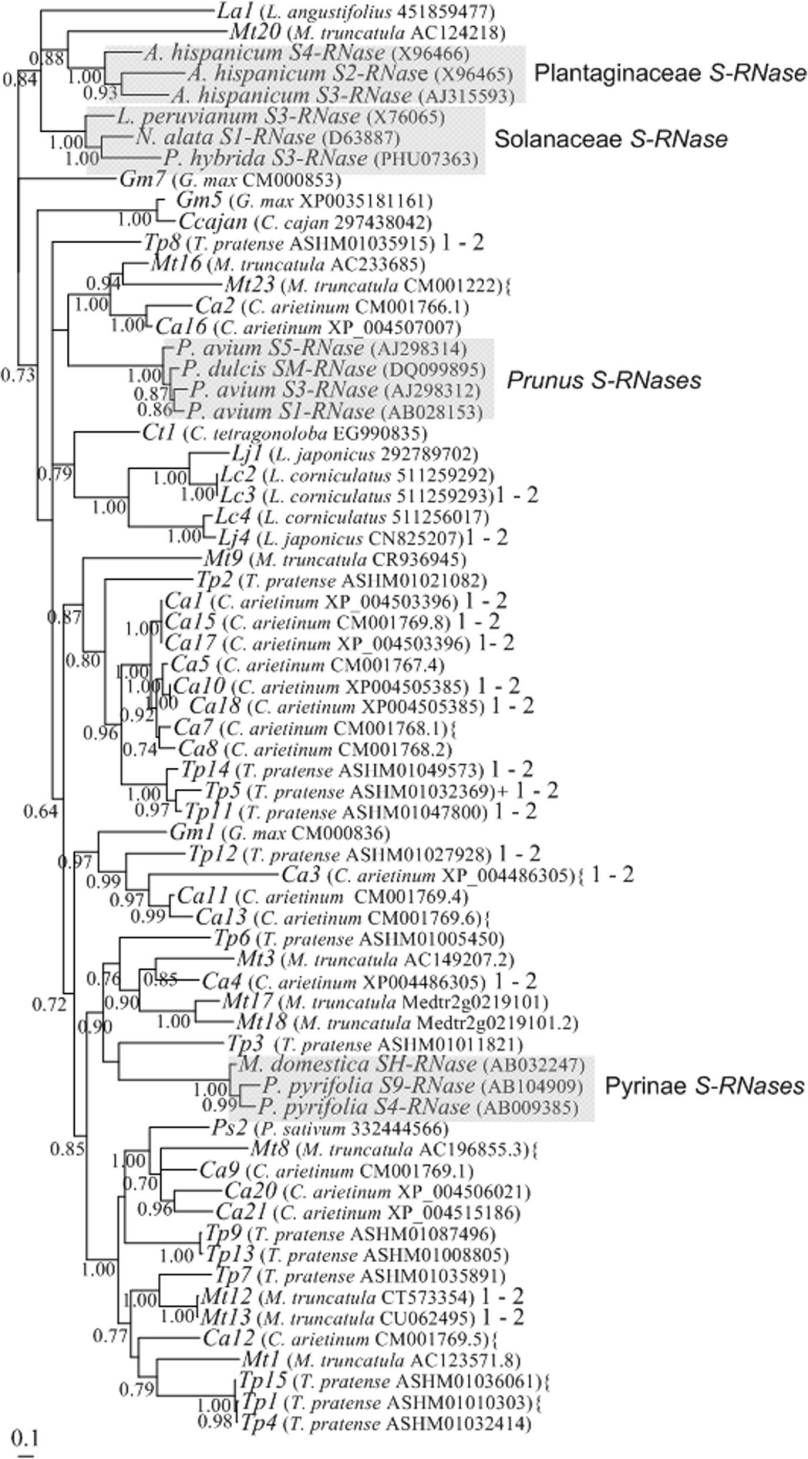
Bayesian phylogenetic tree showing the relationship of the Fabaceae *S-RNase* lineage genes and *Prunus*, Pyrinae, Solanaceae and Plantaginaceae *S-RNases* (shaded sequences). Sequences were aligned using the Muscle algorithm. Numbers below the branches represent posterior credibility values above 60. + indicate the sequences presenting stop codons in the putative coding region. { indicate the sequences where gaps were introduced to avoid stop codons in the putative coding region. The “1 - 2” indicate the sequences presenting amino acid patterns 1 and 2 typical of S-RNases.

Given the evidence for the presence of *RNase* based GSI in Fabaceae (see above), we attempted to identify the *S-RNase* gene in Fabaceae species. Three main criteria were used to first identify putative *S-RNase* lineage genes in the *T. pratense, M. truncatula, C. arietinum, G. max* and *L. angustifolius* genomes, namely: 1) similarity at the amino acid level with *S-RNases* from *Malus* and/or *Prunus* (Methods); 2) the gene must encode a protein where amino acid pattern 4 is absent, once this pattern is found in proteins encoded by non-*S-RNase* lineage genes only [28,29]; and 3) the gene must encode a protein with an isoelectric point higher than 7.5, since S-RNases are always basic proteins [26,30]. Except for *T. pratense*, the genomes here analyzed are from self-compatible species. Nevertheless, the *S*-locus region could also be present, although the *S*-genes could show mutations that disrupt the coding region. For instance, in Rosaceae, mutated versions of the *S-RNase* and/or *SFB* genes have been described in self-compatible species [66]. Table 1 summarizes the features of all gene sequences longer than 500 bp showing similarity at the amino acid level with *S-RNases* from *Malus* and/or *Prunus*. Although intron number was not used as a criterion for the selection of the genes, all these genes have one or two introns in the same location as those of the *S-RNases* [16]. Three *T. pratense* (*TP1*, *Tp5*, and *TP15*, Table 1), two *M. truncatula* (*Mt8* and *Mt23*, Table 1), five *C. arietinum* (*Ca3, Ca6, Ca7, Ca12, Ca13*, Table 1), and one *G. max* (*Gm2*, Table 1) genes are likely non-functional, since they present stop codons in their putative coding region. The number of putative *S*-lineage genes in *T. pratense, M. truncatula, and C. arietinum* (species from the IRLC clade) is about three times larger than in *G. max* (millettioid clade ) or *L. angustifolius* (from the genistoid clade). Although in *C. arietinum* the large number of *T2-RNase* lineage genes can be attributed to recent gene duplications, most of the *T. pratense*, and *M. truncatula* gene duplications are old (Figure 2, and Additional file 2). Three *Lotus corniculatus*, two *L. japonicus*, one *Pisum sativum, one Cajanus cajan, one Lens culinaris, and one Cyamopsis tetragonolobaT2-RNase* sequences that code for putative proteins without amino acid pattern 4, and that code for basic proteins were also included in the phylogenetic analyses (Additional file 3).

According to the phylogenetic analyses, the Fabaceae sequences that show amino acid patterns 1 and 2 (*T. pratense Tp5, Tp8, Tp10, Tp11, Tp12, and Tp14, M. truncatula Mt12 and Mt13, C. arietinum Ca1, Ca3, Ca4, Ca10, Ca15, Ca17, and Ca18, L. corniculatus Lc3, and L. japonicus Lj4*; Table 1 and Additional file 3), that are present in Rosaceae, Solanaceae, Plantaginaceae and Rubiaceae *S-RNases* [28,29], do not cluster toghether (Figure 2, and Additional file 2). Furthermore, Fabaceae genes - *Tp6, Tp3, Ca4, Mt3, Mt17* and *Mt18*, in two of the alignment methods used (Figure 2, and Additional file 2B), cluster with Pyrinae *S-RNases*. *Mt17* and *Mt18* are neighbour genes (they are 3805 bp apart; Table 1). *Mt17* is 56164 bp apart from *Mt3* (Table 1). These genes could also represent the Fabaceae *S-RNase*. Although, the phylogenetic relationship of *M. truncatula Mt20* gene and Plantaginaceae *S-RNases* depends on the alignment method used, we also included this gene in the following analyses.

### Expression patterns of ***T. pratense Tp3***, and ***Tp6***, ***C. arietinum Ca4*** and *M. truncatula Mt3, Mt17, Mt18*, and *Mt20* genes

**Figure 3:**
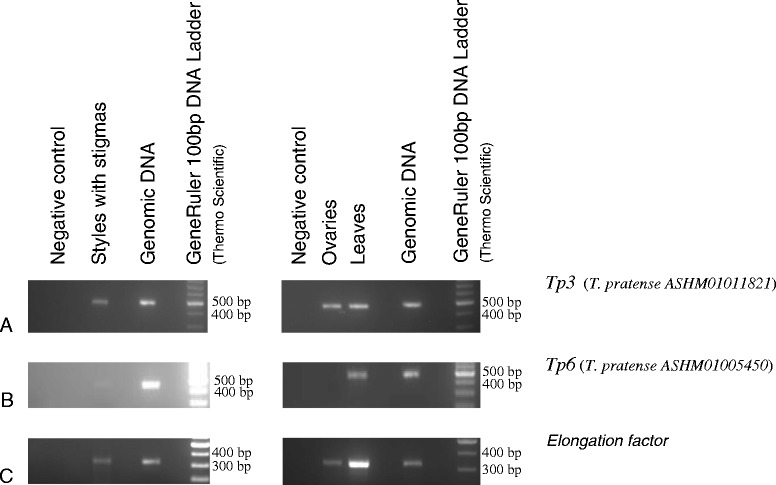
Expression pattern for the *T. pratense Tp3* **(A)**, and *Tp6* **(B)** *S-RNase* lineage genes in pistils, ovaries, and leaves. The elongation factor 1-α (*Elf1-α*) gene, the positive control for cDNA synthesis, is presented for these tissues **(C)**.

*S-RNase* expression is highest in pistils, although it can show lower expression in stigma and styles (CP Vieira, personal communication; see above; and [29-31,67]). For *T. pratense* we address the expression of genes *Tp3*, and *Tp6* using cDNA of styles with stigmas, ovaries, and leaves. *T3* gene shows expression in styles with stigmas, ovaries, and leaves (Figure 3A). For *T6* gene, expression is observed in the styles with stigmas, and in leaves (Figure 3B). Since *T. pratense* is a SI species, these genes are thus, likely not *S-RNases*. Accordingly, levels of silent site (synonymous sites and non-coding positions) diversity for *Tp3* and *Tp6* genes are 0.008 and 0.011, respectively (based on five individuals and a genomic region of 447 bp and 414 bp, respectively). *S-RNases* show levels of silent variability higher than 0.23 [68].

Genes similar to the *S-RNase* but that are not involved in GSI may, in principle, show expression in other tissues. Indeed, *S-RNase* lineage 1 genes in *Malus* (Rosaceae) are expressed in embryo and seeds (Vieira CP, unpublished). This is in contrast to the *S-RNase* gene expression that is restricted to the stigma, styles and pistils of flowers at anthesis [29,30,67]. Therefore, genes showing expression in tissues other than the stigma, styles and pistils of flowers at anthesis are unlikely to be *S-RNases*. For *C. arietinum Ca4* gene, blast searches against NCBI EST database shows that this gene is expressed in etiolated seedlings [GenBank:XM_004486248]). Thus, this gene is likely a gene not involved in GSI.

According to *M. truncatula* Gene Expression Atlas (Material and Methods) *Mt20* ([GenBank:Mtr.49135.1.S1_at]) also shows expression in leaf and root tissues, among other tissues analysed. Since *Mt3, Mt17* and *Mt18* genes are not represented in the Affymetrix GeneChip, used in *M. truncatula* Gene Expression Atlas (Material and Methods), we addressed their expression using blastn and the SRA experiment sets for *M. truncatula* (99 RNA-Seq data sets from SRP033257 study from a mixed sample of *M. truncatula* root knot galls infected with *Meloidogyne hapla* (a nematode)). We find evidence for expression of the three genes in this large RNA-seq data set (Additional file 4). Therefore, according to gene expression, none of these genes seems to be determining pistil GSI specificity.

### F-box genes in the vicinity of the ***C. arietinum Ca4*** and ***M. truncatula Mt3, Mt17, Mt1***, and ***Mt20*** genes

**Figure 4:**
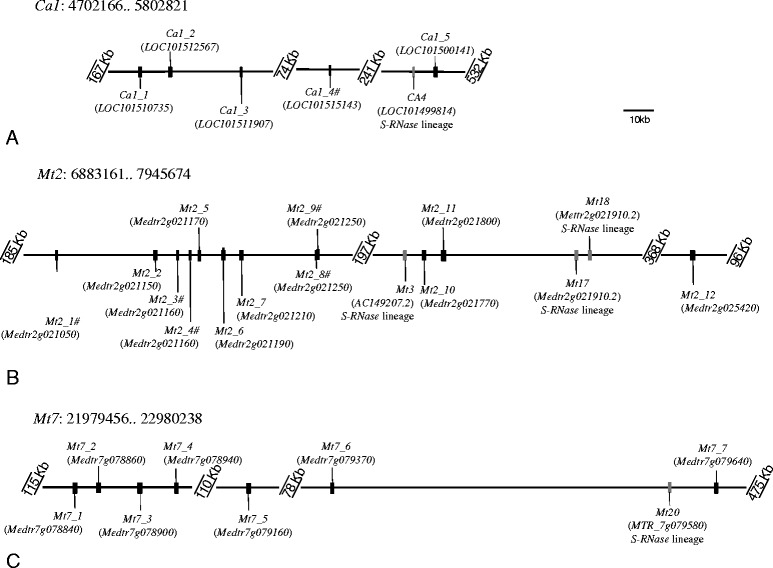
Representation of F-box *SFB* -*SFBB*- and *SLFL*- like genes located in the 500 Kb region surrounding the *C. arietinum Ca4* gene **(A)**, and *M. truncatula Mt3*, *Mt17*, *Mt18* **(B)** *,* and *Mt20 S-RNase* like genes **(C)**, marked in grey. Sequences assigned with # are very divergent sequences that were not included in phylogenetic analyses.

**Figure 5:**
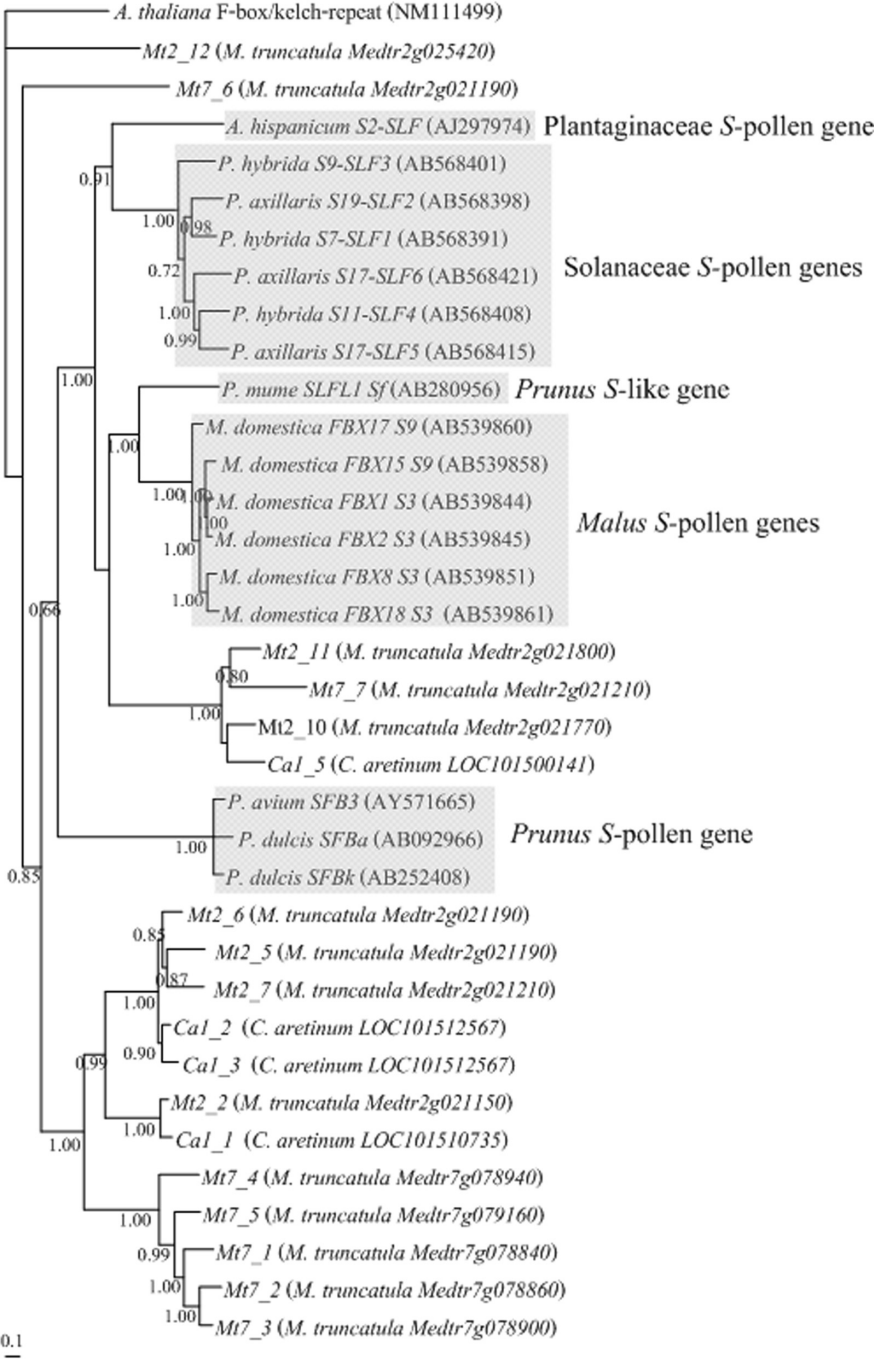
Bayesian phylogenetic tree showing the relationship of the F-box *SFB* -*SFBB*- and *SLFL*- like genes surrounding the *C. arietinum Ca4*, *M. truncatula Mt3*, *Mt17*, *Mt18,* and *Mt20* genes, and *S-*pollen genes from *Prunus*, *Malus*, Solanaceae and Plantaginaceae, and *Prunus S*-like genes (genes not involved in GSI specificity; see Introduction). The reference sequences are shaded. Sequences were aligned using the Muscle algorithm. The tree was rooted using *A. thaliana* F-box/kelch-repeat ([GenBank:NM111499]) gene. Numbers below the branches represent posterior credibility values above 60.

At the *S*-locus region, the *S-RNase* gene is always surrounded by the *S*-pollen gene(s), that can be one gene as in *Prunus* (called *SFB*; [32-37], or multiple genes as in Pyrinae (called *SFBB*s; [38-41,45,47], and Solanaceae (called *SLF*s [14,46,47]). It should be noted that in *Prunus*, other F-box genes called *SLFL*s, not involved in GSI specificity determination [69] are also found surrounding the *S-RNase* gene [32,33]. Therefore, as an attempt to identify the S-locus in Fabaceae species, we identified all *SFBB*s/ *SLF*s, *SLFL*s, and *SFB* like genes in the vicinity (1 Mb) of the *C. arietinum Ca4*, and *M. truncatula Mt3, Mt17, Mt18*, and *Mt20* genes (Figure 4, see Methods). For those gene sequences larger than 500 bp, phylogenetic inferences using reference genes (see Methods) show that *C. arietinum Ca1_5* and *M. truncatula Mt2_10, Mt2_11*, and *Mt7_7* are F-box genes that belong to the *Malus*, Solanaceae, and Plantaginaceae *S*-pollen and *PrunusS*- like pollen genes clade (Figure 5, and Additional file 5).

### Expression pattern of the ***C. arietinum Ca1_5*** and ***M. truncatula Mt2_10, Mt2_11***, and ***Mt7_7*** genes

*Prunus SFB*, *Petunia* and *Antirrhinum SLF*s, and *MalusSFBB* (*S*-pollen genes determining GSI specificity) genes have expression restricted to pollen and anthers [39-41,46,47,70]. Genes showing similarity to *SLF*s but that are not involved in GSI specificity determination (called *SLFL*) have also been described, but they have a broader pattern of expresion. For instance, in *Prunus, SLFL* genes are expressed in pollen and anthers but also in the style [32,33]. Furthermore, in *Malus, SLFL* genes are expressed in pollen, and anthers, but also in pistils, leaves, and seeds (Vieira CP, unpublished). Therefore, we addressed the expression pattern of *C. arietinum Ca1_5* and *M. truncatula Mt2_10, Mt2_11, and Mt7_7* genes.

*C. arietinum Ca1_5* gene is expressed in etiolated seedlings ([GenBank:NW_004515210]), as the *S-RNase* like sequence located in its vicinity. Although we do not know if this gene is also expressed in pollen and anthers, because of its expression in seeds it is likely not involved in GSI. *M. truncatulaMt7_7*, and *Mt2_11* genes, according to Gene Expression Atlas (Material and Methods), are expressed in leafs, petiole, stems, flowers, and roots, among other tissues analyzed (*Mt7_7-Mtr.14778.1.S1_at*, and *Mt2_11 - Mtr.2939.1.S1_at*). For Mt2_11 gene an EST ([GenBank:CA990259.1]) also supports expression of this gene in immature seeds 11 to 19 days after pollination. *Mt2_10* gene is not represented in the Affymetrix GeneChip, and there is no EST data for this gene. Therefore, we addressed their expression using blastn and the SRA SRP033257 experiment data sets for *M. truncatula* (a mixed sample of *M. truncatula* root knot galls infected with *M. hapla*). We find evidence for expression of this gene in this large RNA-seq data set (Additional file 4). Therefore according to gene expression, none of these genes seems to be determining *S*-pollen GSI specificity.

### ***T2-RNases*** from the C. striatus style with stigma transcriptome

Since we found no evidence in the available Fabaceae genomes for *S-RNase* like genes that could be involved in GSI specificity, we performed a transcriptome analysis of *C. striatus* styles with stigmas. This species has been described as having partial GSI [7]. Five *C. striatus* sequences obtained from the style with stigma transcriptome show similarity with *S-RNases* (Table 2; PRJNA279853; http://evolution.ibmc.up.pt/node/77; http://dx.doi.org/10.5061/dryad.71rn0). *CsRNase4*, and *CsRNase5* genes encode proteins with amino acid pattern 4, that is absent from all known S-RNases [28,29]. These genes encode putative acidic proteins (with an isoelectric point of 4.63 and 4.92, respectively), in contrast with S-RNases that are always basic proteins [26,30]. Furthermore, they share at least 85% amino acid similarity with other Fabaceae proteins that are expressed in tissues other than pistils (*G. max* [GenBank:XP_003518732.1], and [GenBank:XP_001235183.1], respectively). Moreover, these genes have three introns, and known *S-RNases* have only one or two introns [16]. Therefore *CsRNase4*, and *CsRNase5* genes are not *S-RNases*.

**Table 2:** ***C. striatus T2- RNases*** present in the style with stigma transcriptome (PRJNA279853; http://evolution.ibmc.up.pt/node/77; http://dx.doi.org/10.5061/dryad.71rn0)

**Gene**	**Transcriptome annotation**	**Size (bp)**	**Amino acid patterns**		
			**1**	**2**	**4**
*CsRNase 1*	c46311_g1	876	FTIHGLWPDN	WPRLFTA	-
*CsRNase 2*	c46642_g1	831	FTIHGLWPD*Y*	WP*S*LS*C*S	*K*PSS*C*N
*CsRNase 3*	c75927_g1	248	FSVHGLWPST	NA	NA
*CsRNase4*	c48285_g2	594	F*G*IHGLWPN*Y*	WP*T*LS*C*P	CPSSNG
*CsRNase5*	c49408_g1	681	F*G*IHGLWPN*Y*	WP*S*LS*C*P	CPSSNG

Underscored are the amino acids that are not allowed in the motifs of [28].

NA- the available sequence does not cover this region.

**Figure 6:**
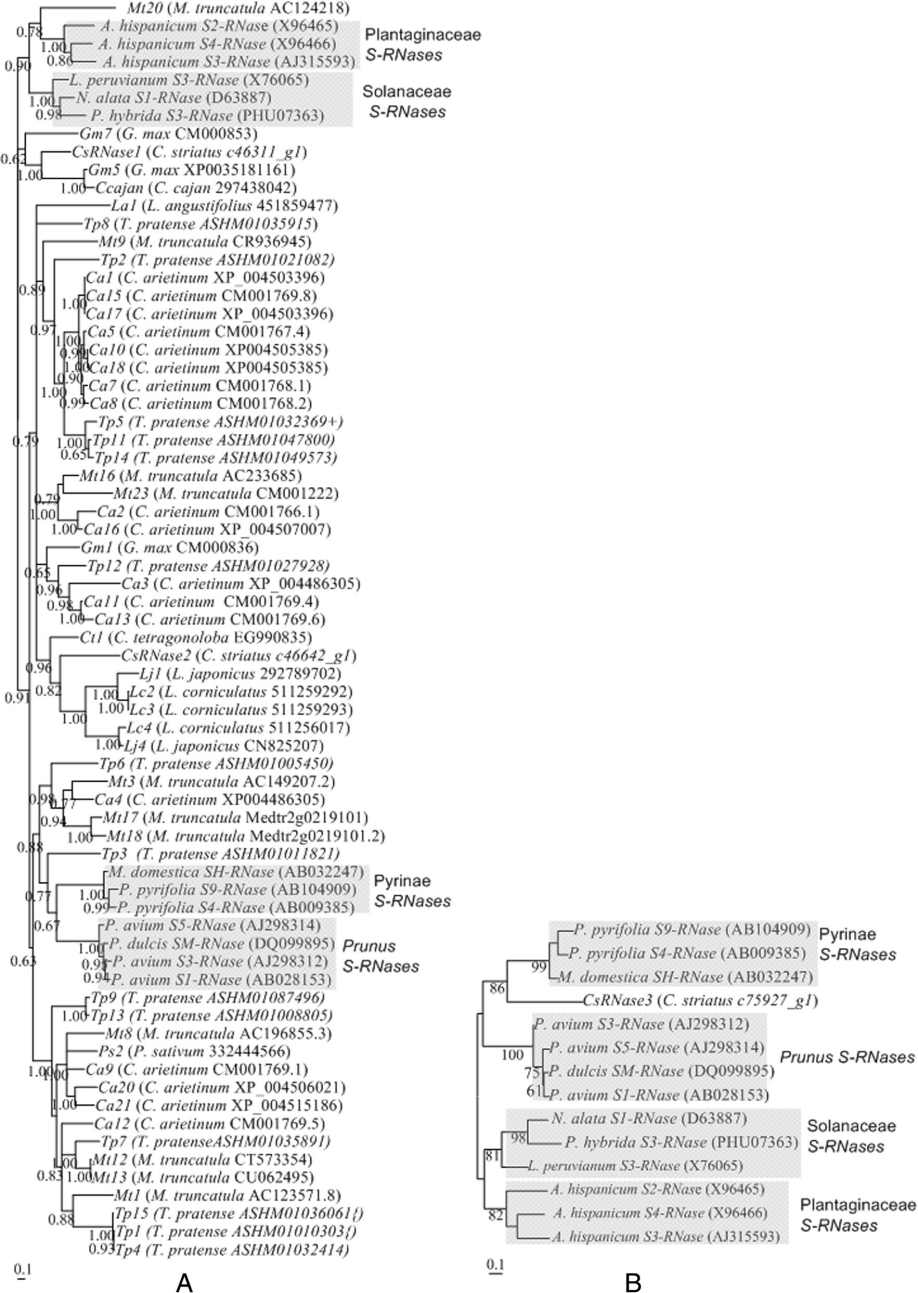
Bayesian phylogenetic trees showing the relationship of: **(A)** *C. striatus CsRNase1*and *CsRNase2* genes and Fabaceae *S-RNase* lineage genes, and *Prunus*, Pyrinae, Solanaceae and Plantaginaceae *S-RNases*. Sequences were aligned using the Muscle algorithm; and **(B)** *CsRNase3* gene and *Prunus*, Pyrinae, Solanaceae and Plantaginaceae *S-RNases*. The reference sequences are shaded. Numbers below the branches represent posterior credibility values above 60.

**Figure 7:**
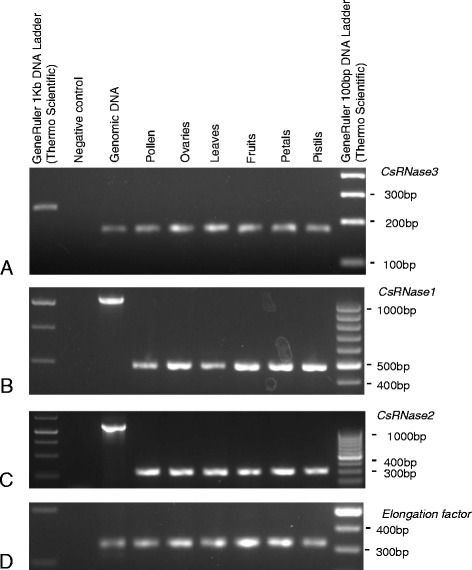
Expression pattern for the *C. striatus S-RNase* lineage genes *CsRNase3* **(A)**, *CsRNase1* **(B)**, and *CsRNase2* **(C)** in pollen, ovaries, leaves, fruits, petals and pistils. The elongation factor 1-α (*Elf1-α*) gene, the positive control for cDNA synthesis, is presented for these tissues **(D)**.

*CsRNase1*, and *CsRNase2* genes code for proteins that do not present amino acid pattern 4, like the *S-RNase* gene (Table 2). Because the *CsRNase3* coding sequence is incomplete, it is not possible to ascertain whether the protein encoded by this gene shows the amino acid pattern 4. Phylogenetic analyses of *CsRNase1*, and *CsRNase2* genes, together with the sequences of other Fabaceae *S*-lineage genes, Rosaceae, Solanaceae, and Plantaginaceae *S-RNases* show that none of these genes belong to the known *S-RNase* gene lineages (Figure 6A, Additional file 6). *CsRNase3* gene, however, clusters with Pyrinae *S-RNases*, and thus could represent a putative *S-RNase* gene (Figure 6B). For *CsRNase3* gene, in the 266 bp region available, there are no introns. Accordingly, in the corresponding region there are no introns at the *S-RNase* gene. Nevertheless, unlike the *S-RNases*, *CsRNase3* gene is expressed in ovaries, petals, leaves and fruits (Figure 7A). Moreover, levels of silent site diversity for this gene are moderate (π = 0.0233; based on a genomic region of 133 bp and five individuals of *C. striatus* from the Marecos population), but lower than that of the *S-RNase* gene (higher than 0.23; [68]). Thus, *CsRNase3* gene does not present the expected features of a *S-RNase* gene.

Since we could not find any *S-RNase* candidate belonging to the Rosaceae, Solanaceae and Plantaginaceae *S-RNase* lineage genes, we characterized the *CsRNase1* and *CsRNase2* genes, that do not belong to any of the known *S-RNases* lineages. *CsRNase1* gene is one of the most expressed genes (see Fragments Per Kilobase of target transcript length per Million reads mapped (FPKM) at position 24 in Additional file 7), but their genomic sequence revealed three introns (Additional file 8A). Moreover, *CsRNase1* gene is expressed in ovaries, petals, pistils, leaves and fruits (Figure 7B), in contrast with the *S-RNases* that are expressed mainly in pistils [29,30,67]. Furthermore, levels of silent site (synonymous sites and non-coding positions) variability for this gene are low (π = 0.0006; based on a genomic region of 1020 bp and five individuals of *C. striatus* from the Marecos population) which is in sharp contrast with the expectation of high levels of variability at the *S-RNase* gene [68]. Therefore, the overall evidence is that the *CsRNase1* gene is not a *S*-pistil gene. For *CsRNase2* gene the genomic sequence revealed five introns (Additional file 8B), it shows expression in ovaries, petals, pistils, leaves and fruits (Figure 7C), and low levels of silent site (synonymous sites and non-coding positions) variability (π = 0.0157; based on a genomic region of 1147 bp and five individuals of *C. striatus* from the Marecos population). Therefore, *CsRNase2* gene is also not a *S*-pistil gene.

## Discussion

Phylogenetic analysis of *T2-RNase* genes from five Fabaceae genomes and one pistil transcriptome revealed more than six *S*-lineage genes. The two *T. pratense* genes that are phylogenetically related with Pyrinae *S-RNases* show, however, expression and polymorphism levels incompatible with being involved in GSI. Although the breeding system of the *T. pratense* individuals used in the polymorphism analyses was not characterized, in the literature all individuals analysed are SI [11,71,72]. Furthermore, red clover is described as being difficult to self, because of low seed set after selfing [72]. Furthermore, the sequences obtained for the Portuguese population for the two *T. pratense* genes phylogenetically related with Pyrinae *S-RNases*, are very similar to those of the individual used for the *T. pratense* genome. Furthermore, none of the Fabaceae *T2-RNase* genes phylogenetically related with known *S-RNases*, revealed expression patterns compatible with a candidate Fabaceae *S*-pistil gene. It could be argued that only *T. pratense* is a self-incompatible species [71,73], and that the *S*-locus region may not be present in the other available genomes. Nevertheless, the presence of the same gene lineages in the *T. pratense, M. truncatula* and/or *C. arietinum* suggests that this is not the case. In Rosaceae, SC species still present the *S*-locus region, but *S-RNase* and *SFB* genes are non-functional [66]. Nevertheless, mutations at loci involved in GSI but that are unlinked to the S-locus are also observed [74,75]. A similar pattern is also described in other SI systems such as that present in Brassicaceae family. For instance, the *S*-locus is present in the genome of the SC *Arabidopsis thaliana*, but the genes determining *S*-specificity are non-functional [76,77]. It should be noted, however, that the SI loss in *M. truncatula* is at least twice as old as that of *A. thaliana*. Therefore, genomes of SC species can also help in the identification of the putative *S*-locus genes.

The presence of Fabaceae sequences that cluster with Pyrinae *S-RNases* and *S*-pollen genes supports the hypothesis that we have identified the orthologous Pyrinae *S*-locus region in Fabaceae. These genes in Fabaceae seem to be performing functions other than GSI. Nevertheless, to exclude these genes as being the ones determining GSI, segregation analyses from controlled crosses are needed to show that these genes do not segregate as *S*-locus genes.

The fact that in Fabaceae, the Rosaceae, Solanaceae, and Plantaginaceae *S-RNase* gene lineages seem not to be involved in GSI, raises the hypothesis that in *Trifolium* GSI could be not *RNase* based. This hypothesis has been suggested before, based on the observation that on *M. truncatula* chromosome 1, that is largely syntenic to linkage group HG1 of *T. pratense*, where the *S*-locus has been mapped, there are no *T2-RNases* exhibiting significant similarity to Solanaceae, Rosaceae and Plantaginaceae *S-RNases*. The same observation has been reported for the numerous *T2-RNase* like sequences in the *M. truncatula* genome, even for those located near F-box genes, like the *S-RNases* [9]. Nevertheless, under the current hypothesis, *RNase* based GSI evolved only once [26-28]. It is, however, conceivable that the ancestral *S*-locus has been duplicated during evolution. The presence of Fabaceae sequences presenting motifs 1 and 2 along the phylogeny support this hypothesis. In *C. striatus*, however, none of the *T2-RNase* genes expressed in pistils is determining GSI. Thus, there is no evidence to suggest that other *T2-RNase* lineage genes could be determining Fabaceae GSI. If this is the case, Fabaceae GSI has evolved the novo from *T2-RNase* unrelated genes, and thus, the information on Solanaceae, Rosaceae, Plantaginaceae and Rubiaceae *S-RNases* is not useful for the identification of the Fabaceae *S*-locus. It is expected that the *S*-pistil gene is highly expressed in the tissue where GSI occurs, and transcriptome analyses of this tissue can produce a list of genes showing high expression levels, such as that we present for *C. striatus* (see Additional file 7). Nevertheless, expression analyses, levels of diversity, and segregation analyses in controlled crosses will be needed to identify which gene(s) is(are) involved in *S*-pistil specificity.

It should be noted that in several Fabaceae species, we find *SSK1* like genes with the typical features of those found in *S-RNase* based SI species from other plant families. It is conceivable that *SSK1* like genes will be present in species where *T2-RNase* genes belonging to the *S*-lineage are present, even though such genes may not be involved in *RNase* based GSI. This must be the case for *C. striatus*. Moreover, the presence of a *SSK1* like gene in *C. clementina* where no *T2-RNases* were identified from the transcriptome analyses of stylar cells of a self-incompatible and a self-compatible cultivar [63] offers support to this hypothesis.

The possibility that the frequency of self-incompatible species is overestimated in Fabaceae should not be also, ruled out. Indeed, the presence of binucleate pollen (typically associated with GSI), as well as fruit and seed production, are frequently used to assess the breeding system of a species. Nevertheless, other processes known to occur in Fabaceae species can affect fruit and seed production. For instance, Papilionoideae species have a membrane at the stigmatic surface that needs to be disrupted for pollen grain germination. In species of this subfamily the flower’s own pollen can cover the stigma at the bud stage [7,78-81], but it does not germinate while the stigmatic surface is intact [5,7,82]. With flowering maturation this stigmatic membrane in SI species must be scratched by a pollinator that visits the flower [7,19,83]. Moreover, late-acting self-incompatibility (LSI) has been described in many Fabaceae species such as *Medicago sativa* [84], *Vicia faba* [17], *Pisum sativum* [22], and *Colutea arborescens* [19] from the IRLC clade; *Lotus corniculatus* [85] and *Coronilla emerus* [19], both from the robinoid clade; *Phaseolus vulgaris* [23] from the millettioid clade; *Dalbergia miscolobium* [82] and *D.retusa* [86] from the dalbergioid clade; as well as in *Genista hirsuta, Adenocarpus complicatus, Retama sphaerocarpa,Cytisus striatus, C. grandiflorus* [7,83], and *C. multiflorus* [83,87] from the genistoid clade. In Fabaceae, LSI is due to multiple causes such as disharmony in endosperm/embryo development [87], differential growth rate of the pollen tubes within the ovaries [18,24], embryonic abortion [22,23] and inbreeding depression [83]. Although the genetics and physiology of LSI is still poorly understood, it is clear that it can be genetically determined [21], and that LSI and GSI can co-occur, as it happens in *C. striatus* [7]. Indeed, LSI implies similar growth of pollen tubes in the style following self- and cross-pollination (see for instance [88,89]), and in this species there is a significant difference in the percentage of pollen growth in self and cross-pollinations. Therefore, besides LSI, an additional partial GSI system has been inferred in *C. striatus* [7]. Similar inferences have been made for *V. faba* [17], *L. corniculatus* [18], *C. emerus* and *Colutea arborescens* [19].

## Conclusion

There is no evidence for Rosaceae, Solanaceae, and Plantaginaceae S-RNase lineage genes determining GSI in Fabaceae species. LSI is frequent in this family and may co-occur with GSI. Nevertheless, so far, in Fabaceae, only *Trifolium* species have been described as presenting GSI only. Thus, LSI or LSI in combination with GSI, will be likely the major hurdle when attempting to efficiently incorporating traits of agronomical interest from wild populations into crop varieties.

## Methods

### ***SSK1*** like genes

To identify *SSK1* like sequences in flowering plants we have used NCBI’s Pattern hit initiated blastp using as query *A. hispanicum SSK1* ([GenBank:ABC84197.1]) and the pattern WAFExxxxD, as well as *Pyrus x bretschneideri SSK1* like ([GenBank:CCH26218.1]), and *Prunus avium SSK1* like ([GenBank:AFJ21661.1]) proteins and the pattern GVDED. For the non-annotated *T. pratense* genome ([GenBank:PRJNA200547]; [72]) we have obtained all putative open reading frames longer than 100 bp (getorf; http://emboss.sourceforge.net; [90]). Then we used local tblastn [91], with an Expect value of (e) < 0.05, and as query the above Rosaceae SSK1 like proteins.

### *T. pratense, M. truncatula, C. arietinum, G. max and L. angustifoliusS*-RNase lineage genes

Since four out of the five genomes here studied are from self-compatible species, *S*-pistil genes may be present as non-annotated pseudogenes. Therefore, putative open reading frames longer than 100 bp (getorf; http://emboss.sourceforge.net; [90]) were obtained for *T. pratense* ([GenBank:PRJNA200547]; [72]), *M. truncatula* ([GenBank:PRJNA30099], [GenBank:PRJNA10791], [92]; http://www.medicagohapmap.org), *C. arietinum* ([GenBank:PRJNA190909], [GenBank: PRJNA175619], [93]; http://cicar.comparative-legumes.org), *G. max* ([GenBank: PRJNA483899], [GenBank:PRJNA19861], [94]; http://www.Soybase.org) and *L. angustifolius* ([GenBank:PRJNA179231]; [95]; http://lupinus.comparative-legumes.org) genomes. Then, *T2-RNase* lineage sequences (including putative pseudogenes) of these species were identified and annotated by homology using local tblastn [91], using an Expect value of (e) < 0.05, and as query the *M. domesticaS2-RNase* ([GenBank:AAA79841.1]), and *P. persicaS1-RNase* ([GenBank:BAF42768.1]) proteins. If the inferred genes have been annotated before, the original name and accession number is indicated for that gene. Only sequences larger than 500 bp, and not presenting pattern 4 (absent in all S-RNases; [28]), were considered. In some cases, sequences were curated by introduction of sequence gaps to extend recognizable homology with the query sequence. Other Fabaceae *T2-RNase* sequences from *M. sativa, Pisum sativum, Lens culinaris*, (also belonging to IRLC), *Lotus corniculatus, L. japonicus* (from the robinoid clade), *Cajanus cajan*, (from the millettiod clade), *Cyamopsis tetragonoloba* (from the indigoferoid clade), and *Arachis hypogaea* (from the dalbergioid clade) were obtained from GenBank, using tblastn, an Expect value (e) < 0.05, and the above *M. domestica*, and *P. persica* sequences as query (Additional file 3). For all peptides, isoelectric points were calculated using ExPASy [96]. Given the large number of genes analysed, for the sake of simplicity, in this work, we use short gene codes rather than the long mostly non-informative gene names. The correspondences between gene codes and gene names are given in Table 1, and Additional file 3.

### F-box *SFBB* - and *SFB* - like genes in the vicinity of *C. arietinum* and *M. truncatulaS****-RNase*** like genes

Putative open reading frames longer than 100 bp (getorf; http://emboss.sourceforge.net; [90]) were obtained for the 500 Kb of the *C. arietinum* and *M. truncatula* regions surrounding putative *S-RNase* lineage genes. F-box genes were identified and annotated by homology using local tblastn [91], an Expect value of (e) < 0.05), and the *M. domesticaSFBB3-beta* ([GenBank:AB270796.1]), *P. avium SFB3* ([GenBank:AY571665.1]), and *P. axillarisS19-SLF* ([GenBank:AY766154.1]) proteins. The correspondences between gene codes and gene names are given in Additional file 9.

### Phylogenetic analyses

Five data sets were used: 1- *SSK1* like genes from flowering plants (that includes as reference sequences from Solanaceae, Plantaginaceae and Rosaceae *SSK1* like genes), 2- Fabaceae *S-RNase* like genes that encode proteins with an isoelectric point higher than 7.5 (S-RNases are always basic proteins; [26]), with the exception of the *Mt5, Mt26, Ca14* and *Ca19* sequences that result in the introduction of many alignment gaps in the resulting alignment. Reference sequences are Solanaceae, Plantaginaceae and Rosaceae *S-RNase* genes, 3- *C. arietinum* and *M. truncatula* F-box *SFBB* - and *SFB* - like genes in the vicinity of *S-RNase* lineage genes. Reference sequences are Solanaceae and Plantaginaceae *SLF*s, *Malus SFBBs* and *Prunus SFB*, and Rosaceae *S*-pollen like genes (genes similar to *S*-pollen genes but that are not involved in GSI specificity), 4- *C. striatus CsRNase1*, and *CsRNase2* genes. Reference sequences are Fabaceae *S-RNase* like genes that encode proteins with an isoelectric point higher than 7.5, Solanaceae, Plantaginaceae and Rosaceae *S-RNase* genes, and 5- *C. striatus CsRNase3* gene. Reference sequences are Solanaceae, Plantaginaceae and Rosaceae *S-RNase* genes. With the exception of data set 5 (because of the size (264 bp) of *C. striatusCsRNase3* sequence), sequences in the data sets were aligned with the ClustalW2, Muscle and T-coffee alignment algorithms as implemented in ADOPS [97]. Only codons with a support value above two are used for phylogenetic reconstruction. Bayesian trees were obtained using MrBayes 3.1.2 [98], as implemented in the ADOPS pipeline, using the GTR model of sequence evolution, allowing for among-site rate variation and a proportion of invariable sites. Third codon positions were allowed to have a gamma distribution shape parameter different from that of first and second codon positions. Two independent runs of 2,000,000 generations with four chains each (one cold and three heated chains) were set up. The average standard deviation of split frequencies was always about 0.01 and the potential scale reduction factor for every parameter about 1.00 showing that convergence has been achieved. Trees were sampled every 100th generation and the first 5000 samples were discarded (burn-in). The remaining trees were used to compute the Bayesian posterior probabilities of each clade of the consensus tree.

In the phylogenetic analyses that include *C. striatusCsRNase3* gene we used the MEGA 5 software [99]. The alignment was performed using ClustalW, and for the phylogenetic reconstruction we used pairwise deletion and minimum evolution method. We run 10000 bootstrap replications, using maximum composite likelihood method, and including transitions + transversions substitutions, and all codons.

### Expression of *T. pratense Tp3* and *Tp6* genes in styles with stigmas, ovaries, petals and leaves

To collect enough material for the cDNA synthesis of style with stigma (since in *T. pratense* each individual has less than three inflorescences with less than 50 flowers at anthesis), we have mixed the plant material obtained from two different individuals. These individuals present an amplification product of the expected size, obtained from genomic DNA (extracted from leaves, using the method of Ingram et al. [100]), using specific primers for *Tp3* and *Tp6* genes (Additional file 10), and standard amplification conditions of 35 cycles of denaturation at 94°C for 30 s, primer annealing temperature according to Additional file 10 for 30 s, and primer extension at 72°C for 2 min. More than 500 styles with stigmas were collected from these two individuals, that were frozen in liquid nitrogen and stored at −80°. For one of these individuals we also collected ovaries, and leaves. Total RNA was extracted using TRIzol® (Invitrogen, Spain) according to the manufacturer’s instructions and treated with DNase I (Turbo RNase-Free) (Ambion, Portugal). RNA quantity was assessed by NanoDrop v.1.0 (Thermo Scientific). cDNA was synthesized with SuperScript® III First-Strand Synthesis System for RT-PCR from Invitrogen. *Elongation factor1-α* (*Elf1-α*) was used as positive control for cDNA synthesis. Standard amplification conditions as described above were used.

### Levels of diversity at *T. pratense Tp3* and *Tp6* genes

To determine levels of diversity for *Tp3* and *Tp6* genes, genomic DNA from leaves of five *T. pratense* individuals of a Porto population (assigned as TpPorto1to TpPorto5) was extracted using the method of Ingram et al. [100]. For each individual, genomic DNA was used in PCR reactions using primers 1821 F + 1821R, and 5450 F + 5450R, to amplify *Tp3* and *Tp6* genes, respectively (Additional file 10). Standard amplification conditions were 35 cycles of denaturation at 94°C for 30 seconds, primer annealing according to supplementary Additional file 10 for 30 s, and primer extension at 72°C for 3 min. The amplification products were cloned, using the TA cloning kit (Invitrogen, Carlsbad, CA). For each amplification product, the insert of an average of 10 colonies was cut separately with RsaI, and Sau3AI restriction enzymes. For each restriction pattern three colonies were sequenced in order to obtain a consensus sequence. The ABI PRISM BigDye cycle-sequencing kit (Perkin Elmer, Foster City, CA), and specific primers, or the primers for the M13 forward and reverse priming sites of the pCR2.1 vector, were used to prepare the sequencing reactions. Sequencing runs were performed by STABVIDA (Lisboa, Portugal). DNA sequences were deposited in GenBank (accession numbers KR054719 - KR054728). Nucleotide sequences were aligned using ClustalW algorithm as implemented in MEGA 5 [99]. Analyses of DNA polymorphism were performed using DnaSP (version 4.1) [101].

### Expression of *M. truncatula Mt3, Mt17, Mt18, Mt20*, *Mt7_7, Mt2_10*, and *Mt2_11* genes

For the genes of interest, using blast at *M. truncatula* gene expression atlas (http://mtgea.noble.org/v3/; Affymetrix GeneChip Medicago Genome Array; [102]) we identify Probeset ID and the expression pattern associated with that probe. For the genes not represented in the *M. truncatula* gene expression atlas, we used blastn and the SRA SRP033257 experiment sets for *M. truncatula* (99 RNA-Seq data sets from a mixed sample of *M. truncatula* root knot galls infected with *Meloidogyne hapla* (a plant-nematode)).

### *Cytisus striatus* style with stigma transcriptome

*C. striatus* has been described as having partial GSI, since a fraction (about 27%) of self-pollen tubes after hand self-pollination, stop growing along the style and the ovary [7]. For one *C. striatus* individual (assigned as Cs1), from a population at Marecos (Valongo, Portugal), 400 flower buds ranging from 1.8 to 2 cm (the size of pre-anthesis stages; [103] were dissected to collect the styles with stigmas, that were frozen in liquid nitrogen and stored at −80°. Total RNA was extracted as described above. RNA quantity was assessed by NanoDrop v.1.0 (Thermo Scientific) and RNA quality by BioRad’s Experion System. A total RNA sample of approximately 2.691 μg ,with RQI of 7.1, and a 260/280 nm absorption ratio 2.08 was obtained. Total RNA was processed for Illumina RNA-Seq, at BGI (Hong Kong, China).

Only high quality reads were provided by BGI. Before assembly, adaptor sequences were removed from raw reads. FASTQC reports were then generated and based on this information the resulting reads were trimmed at both ends. Nucleotide positions with a score lower than 20 were masked (replaced by an N). These analyses were performed using the FASTQ tools implemented in the Galaxy platform [104-106]. The resulting high-quality reads were used in the subsequent transcriptome assembly using Trinity with default parameters [107]. The Transcriptome project has been deposited at GenBank PRJNA279853, and the assembled transcriptome at http://evolution.ibmc.up.pt/node/77, or http://dx.doi.org/10.5061/dryad.71rn0. All contigs were used as queries for tblastn searches using local blast [91], and the *SSK1* and *S-RNase* query sequences reported above. Fragments Per Kilobase of target transcript length per Million reads mapped (FPKM) values were estimated using the eXpress software [108] as implemented in Trinity. BLAST2Go [109] was used to determine PFAM (protein families) codes for the 100 most expressed genes.

### The genomic sequence of the *C. striatusS*-lineage *T2-RNases*

 To determine intron number for *C. striatus CsRNase1*, *CsRNase2*, and *CsRNase3*, primers were designed (Additional file 10) based on the sequences obtained from the transcriptome. Genomic DNA was extracted from leaves of the Cs1 individual, as described above, and used as template in PCR reactions. Standard amplification conditions were 35 cycles of denaturation at 94°C for 30 seconds, primer annealing according to Additional file 10 for 30 s, and primer extension at 72°C for 3 min. The amplification products were cloned, and sequenced as described above. The genomic sequences for *C. striatus CsRNase1* and *CsRNase2* genes of individual Cs1 were deposited at GenBank (accession numbers KR054703, and KR054709).

### Expression of the *C. striatusS*-lineage *T2-RNase* genes in pollen, ovaries, petals, pistils, leaves and fruits

Pollen, ovaries, petals, pistils, leaves and fruits from individual Cs1 were collected and immediately frozen in liquid nitrogen and stored at −80°. Total RNA and cDNA synthesis was performed as described above. *Elongation factor1-α(Elf1-α*) was used as positive control for cDNA synthesis. Primers CytSRN-62 F + CytisusRNase531R, CytR2-cons142F + CytR2-445R, and Cy10F + Cy10R were used for the amplification of the *CsRNase1*, *CsRNase2*, and *CsRNase3* genes, respectively (Additional file 10). Standard amplification conditions were 35 cycles of denaturation at 94°C for 30 s, primer annealing temperature according to Additional file 10 for 30 s, and primer extension at 72°C for 2 min.

### Nucleotide diversity at ***C. striatus****S*-lineage genes

To determine levels of diversity for *CsRNase1*, *CsRNase2*, and *CsRNase3* genes, genomic DNA from leaves of four *C. striatus* individuals of the Marecos population (assigned as Cs2 to Cs5) was extracted as described above. For each individual, genomic DNA was used in PCR reactions using the same primers and conditions described above. The amplification products were cloned, as described above. For each amplification product, the insert of an average of 10 colonies was cut separately with *Rsa*I, and *Sau*3AI restriction enzymes. For each restriction pattern three colonies were sequenced in order to obtain a consensus sequence. Sequencing has been performed as described above. DNA sequences were deposited in GenBank (accession numbers KR054704 - KR054707, KR054710 - KR054713, and KR054714 - KR054718, respectively). Nucleotide sequences were aligned using ClustalW algorithm as implemented in MEGA 5 [99]. Analyses of DNA polymorphism were performed using DnaSP (version 4.1) [101].

### Availability of supporting data

The *C. striatus* assembled transcriptome, supporting the results of this article is available in the [*Cytisus striatus* style with stigma transcriptome] repository [http://evolution.ibmc.up.pt/node/77], and at Dryad [http://dx.doi.org/10.5061/dryad.71rn0].

The data used to perform the phylogenetic analyses is available at Dryad [http://dx.doi.org/10.5061/dryad.71rn0].

## Additional files

Additional file 1:
**Bayesian phylogenetic trees showing the relationship of** ***SSK1*** **like genes in flowering plants.** Sequences were aligned using ClustalW2 (A), and T-coffee (B) algorithms. The tree was rooted using *O. sativa* ([GenBank:AP003824]) and *C. maxima* ([GenBank:FJ851401]) genes. Numbers below the branches represent posterior credibility values above 60. Additional file 2:
**Bayesian phylogenetic trees showing the relationship of Fabaceae** ***S-RNase*** **lineage genes and** ***Prunus*** **, Pyrinae, Solanaceae and Plantaginaceae** ***S-RNases.*** Sequences were aligned using ClustalW2 (A), and T-coffee (B) algorithms. The reference sequences are shaded. Additional file 3:
**Fabaceae** ***T2-RNases*** **available in GenBank not presenting amino acid pattern 4.**
Additional file 4:
**Reads from the SRP033257 experiment of** ***M. truncatula*** **(RNA-Seq data sets from a mixed sample of** ***M. truncatula*** **root knot galls infected with** ***Meloidogyne hapla*** **(a plant-nematode)) supporting the expression of the** ***Mt3*** **,** ***Mt17*** **,** ***Mt18*** **,** **and** ***Mt_10*** **genes.**
Additional file 5:
**Bayesian phylogenetic trees showing the relationship of the F-box** ***SFB*** **-** ***SFBB*** **- and** ***SLFL*** **- like genes surrounding the** ***C. arietinum Ca4*** **,** ***M. truncatula Mt3*** **,** ***Mt17*** **,** ***Mt18*** **,** **and** ***Mt20*** **genes, and** ***S-*** **pollen genes from** ***Prunus*** **,** ***Malus*** **, Solanaceae and Plantaginaceae, and** ***Prunus S*** **-like genes (shaded sequences).** Sequences were aligned using ClustalW2 (A), and T-coffee (B) algorithms. The tree was rooted using *A. thaliana* F-box/kelch-repeat ([GenBank:NM111499]) gene. Numbers below the branches represent posterior credibility values above 60. Additional file 6:
**Bayesian phylogenetic trees, showing the relationship of the** ***C. striatus CsRNase1*** **and** ***CsRNase2*** **genes with Fabaceae** ***S-RNase*** **lineage genes and** ***Prunus*** **, Pyrinae, Solanaceae and Plantaginaceae** ***S-RNases*** **(shaded sequences).** Sequences were aligned using ClustalW2 (A), and T-coffee (B) algorithms. Numbers below the branches represent posterior credibility values above 60. Additional file 7:
**The 100 most expressed genes of the** ***C. striatus*** **stigma with style transcriptome.**
Additional file 8:
**Representation of the genomic region of** ***C. striatus CsRNase 1*** **(A) and** ***CsRNase 2*** **(B) genes.** Lines represent introns, grey boxes represent exons, and arrows indicate the most external primers used. Additional file 9:
**Correspondences between gene codes and gene names for F-box** ***SFBB-*** **and** ***SFB*** **- like genes in the vicinity of** ***C. arietinum*** **and** ***M. truncatula S-RNase*** **like genes.**
Additional file 10:
**Primers used in this work.**

## Abbreviations

MYA: Million years ago
SC: Self-compatible
SI: Self-incompatible
GSI: Gametophytic self-incompatibility
IRLC: Inverted-repeat-lacking clade
FPKM: Fragments per kilobase of target transcript length per million reads mapped
IP: Isoelectric point (IP)

## References

[1] De Nettancourt D (1977). Incompatibility in angiosperms.

[2] Arroyo MTK, Armesto JJ, Villagran C (1981). Plant phenological patterns in the high andean cordillera of central chile. J Ecol.

[3] Atwood SS (1940). Genetics of cross-incompatibility among self-incompatible plants of *Trifolium Repens*. J Am Soc Agron.

[4] Heslop-Harrison J, Heslop-Harrison Y (1982). Pollen-stigma interaction in the leguminosae: constituents of the stylar fluid and stigma secretion of *Trifolium pratense* L. Ann Bot.

[5] Shivanna K, Owens S: Pollen-pistil interactions (Papilionoideae). Advances in Legume Biology: Monograph of Systematic Botany Missouri: Missouri Botanical Garden 1989;(29):157–182.

[6] Weller S, Donoghue M, Charlesworth D (1995). The evolution of self-incompatibility in flowering plants: a phylogenetic approach. Experimental and molecular approaches to plant biosystematics.

[7] Rodríguez-Riaño T, Ortega-Olivencia A, Devesa JA (1999). Reproductive biology in two Genisteae (Papilionoideae) endemic of the western Mediterranean region: *Cytisus striatus* and *Retama sphaerocarpa*. Can J Bot.

[8] Igic B, Lande R, Kohn JR (2008). Loss of self‐incompatibility and its evolutionary consequences. Int J Plant Sci.

[9] Casey NM, Milbourne D, Barth S, Febrer M, Jenkins G, Abberton MT (2010). The genetic location of the self-incompatibility locus in white clover (*Trifolium repens* L.). Theor Appl Genet.

[10] Kao TH, Tsukamoto T (2004). The molecular and genetic bases of S-RNase-based self-incompatibility. Plant Cell.

[11] Leduc N, Douglas G, Monnier M, Connolly V (1990). Pollination in vitro: effects on the growth of pollen tubes, seed set and gametophytic self-incompatibility in *Trifolium pratense* L. and *T. repens* L. Theor Appl Genet.

[12] Tao R, Iezzoni AF (2010). The S-RNase-based gametophytic self-incompatibility system in *Prunus* exhibits distinct genetic and molecular features. Sci Hortic (Amsterdam).

[13] De Franceschi P, Dondini L, Sanzol J (2012). Molecular bases and evolutionary dynamics of self-incompatibility in the Pyrinae (Rosaceae). J Exp Bot.

[14] Wang N, Kao TH (2011). Self-incompatibility in *Petunia*: a self/nonself-recognition mechanism employing *S*-locus F-box proteins and S-RNase to prevent inbreeding. Wiley Interdiscip Rev Dev Biol.

[15] Xue Y, Carpenter R, Dickinson HG, Coen ES (1996). Origin of allelic diversity in *Antirrhinum S* locus RNases. Plant Cell.

[16] Vieira C, Charlesworth D (2002). Molecular variation at the self-incompatibility locus in natural populations of the genera *Antirrhinum* and *Misopates*. Heredity.

[17] Rowlands D (1958). The nature of the breeding system in the field bean (*V. faba* L) and its relationship to breeding for yield. Heredity.

[18] Bubar JS (1959). Differences between self-incompatibility and self-sterility. Nature.

[19] Galloni M, Podda L, Vivarelli D, Cristofolini G (2007). Pollen presentation, pollen-ovule ratios, and other reproductive traits in Mediterranean Legumes (Fam. Fabaceae-Subfam. Faboideae). Plant Syst Evol.

[20] Wilkins KA, Poulter NS, Franklin-Tong VE (2014). Taking one for the team: self-recognition and cell suicide in pollen. J Exp Bot.

[21] Allen AM, Hiscock SJ (2008). Evolution and phylogeny of self-incompatibility systems in Angiosperms. Self-incompatibility in flowering plants.

[22] Briggs C, Westoby M, Selkirk P, Oldfield R (1987). Embryology of early abortion due to limited maternal resources in *Pisum sativum* L. Ann Bot.

[23] Sage TL, Webster BD (1990). Seed abortion in *Phaseolus vulgaris* L. Bot Gaz.

[24] Brink R, Cooper D (1938). Partial self-incompatibility in *Medicago sativa*. Proc Natl Acad Sci U S A.

[25] Wikstrom N, Savolainen V, Chase MW (2001). Evolution of the angiosperms: calibrating the family tree. Proc R Soc B - Biol Sci.

[26] Igic B, Kohn JR (2001). Evolutionary relationships among self-incompatibility RNases. Proc Natl Acad Sci U S A.

[27] Steinbachs JE, Holsinger KE (2002). *S*-RNase-mediated gametophytic self-incompatibility is ancestral in eudicots. Mol Biol Evol.

[28] Vieira J, Fonseca NA, Vieira CP (2008). An *S-RNase*-based gametophytic self-incompatibility system evolved only once in eudicots. J Mol Evol.

[29] Nowak MD, Davis AP, Anthony F, Yoder AD (2011). Expression and trans-specific polymorphism of self-incompatibility RNases in *Coffea* (Rubiaceae). PLoS One.

[30] Roalson EH, McCubbin AG (2003). S-RNases and sexual incompatibility: structure, functions, and evolutionary perspectives. Mol Phylogenet Evol.

[31] Vieira J, Ferreira PG, Aguiar B, Fonseca NA, Vieira CP (2010). Evolutionary patterns at the RNase based gametophytic self - incompatibility system in two divergent Rosaceae groups (Maloideae and *Prunus*). BMC Evol Biol.

[32] Ushijima K, Sassa H, Dandekar AM, Gradziel TM, Tao R, Hirano H (2003). Structural and transcriptional analysis of the self-incompatibility locus of almond: identification of a pollen-expressed F-box gene with haplotype-specific polymorphism. Plant Cell.

[33] Entani T, Iwano M, Shiba H, Che FS, Isogai A, Takayama S (2003). Comparative analysis of the self-incompatibility (*S*-) locus region of *Prunus mume*: identification of a pollen-expressed F-box gene with allelic diversity. Genes Cells.

[34] Ikeda K, Igic B, Ushijima K, Yamane H, Hauck N, Nakano R (2004). Primary structural features of the *S* haplotype-specific F-box protein, SFB, in *Prunus*. Sex Plant Reprod.

[35] Sonneveld T, Tobutt KR, Vaughan SP, Robbins TP (2005). Loss of pollen-*S* function in two self-compatible selections of *Prunus avium* is associated with deletion/mutation of an *S* haplotype-specific F-box gene. Plant Cell.

[36] Nunes MD, Santos RA, Ferreira SM, Vieira J, Vieira CP (2006). Variability patterns and positively selected sites at the gametophytic self-incompatibility pollen SFB gene in a wild self-incompatible *Prunus spinosa* (Rosaceae) population. New Phytol.

[37] Vieira J, Santos RA, Ferreira SM, Vieira CP (2008). Inferences on the number and frequency of S-pollen gene (SFB) specificities in the polyploid *Prunus spinosa*. Heredity.

[38] Cheng J, Han Z, Xu X, Li T (2006). Isolation and identification of the pollen-expressed polymorphic F-box genes linked to the *S*-locus in apple (*Malus* × *domestica*). Sex Plant Reprod.

[39] Kakui H, Tsuzuki T, Koba T, Sassa H (2007). Polymorphism of SFBB-gamma and its use for *S* genotyping in Japanese pear (*Pyrus pyrifolia*). Plant Cell Rep.

[40] Sassa H, Kakui H, Miyamoto M, Suzuki Y, Hanada T, Ushijima K (2007). *S locus F-Box brothers*: multiple and pollen-specific F-box genes with *S* haplotype-specific polymorphisms in apple and Japanese pear. Genetics.

[41] Minamikawa M, Kakui H, Wang S, Kotoda N, Kikuchi S, Koba T (2010). Apple *S* locus region represents a large cluster of related, polymorphic and pollen-specific F-box genes. Plant Mol Biol.

[42] De Franceschi P, Pierantoni L, Dondini L, Grandi M, Sansavini S, Sanzol J (2011). Evaluation of candidate F-box genes for the pollen *S* of gametophytic self-incompatibility in the Pyrinae (Rosaceae) on the basis of their phylogenomic context. Tree Genet Genomes.

[43] Kakui H, Kato M, Ushijima K, Kitaguchi M, Kato S, Sassa H (2011). Sequence divergence and loss-of-function phenotypes of *S locus F-box* brothers genes are consistent with non-self recognition by multiple pollen determinants in self-incompatibility of Japanese pear (*Pyrus pyrifolia*). Plant J.

[44] Okada K, Tonaka N, Taguchi T, Ichikawa T, Sawamura Y, Nakanishi T (2011). Related polymorphic F-box protein genes between haplotypes clustering in the BAC contig sequences around the *S-RNase* of Japanese pear. J Exp Bot.

[45] Aguiar B, Vieira J, Cunha AE, Fonseca NA, Reboiro-Jato D, Reboiro-Jato M (2013). Patterns of evolution at the gametophytic self-incompatibility *Sorbus aucuparia* (Pyrinae) *S* pollen genes support the non-self recognition by multiple factors model. J Exp Bot.

[46] Wheeler D, Newbigin E (2007). Expression of 10 S-class *SLF-like* genes in *Nicotiana alata* pollen and its implications for understanding the pollen factor of the S locus. Genetics.

[47] Kubo K, Entani T, Takara A, Wang N, Fields AM, Hua Z (2010). Collaborative non-self recognition system in S-RNase-based self-incompatibility. Science.

[48] Williams JS, Der JP, Kao T-h (2014). Transcriptome analysis reveals the same 17 *S-Locus F-Box* genes in two haplotypes of the self-incompatibility locus of *Petunia inflata*. The Plant Cell Online.

[49] Luu D-T, Qin X, Laublin G, Yang Q, Morse D, Cappadocia M (2001). Rejection of *S*-heteroallelic pollen by a dual-specific S-RNase in *Solanum chacoense* predicts a multimeric SI pollen xomponent. Genetics.

[50] Huang J, Zhao L, Yang Q, Xue Y (2006). AhSSK1, a novel SKP1‐like protein that interacts with the *S*‐locus F‐box protein SLF. The Plant J.

[51] Hua Z, Kao TH (2006). Identification and characterization of components of a putative *Petunia S*-locus F-box-containing E3 ligase complex involved in S-RNase-based self-incompatibility. Plant Cell.

[52] Zhao L, Huang J, Zhao Z, Li Q, Sims TL, Xue Y (2010). The Skp1‐like protein SSK1 is required for cross‐pollen compatibility in *S‐RNase*‐based self‐incompatibility. The Plant J.

[53] Xu C, Li M, Wu J, Guo H, Li Q, Zhang Y (2013). Identification of a canonical SCF^SLF^ complex involved in S-RNase-based self-incompatibility of *Pyrus* (Rosaceae). Plant Mol Biol.

[54] Matsumoto D, Tao R (2012). Yeast Two-Hybrid screening for the general inhibitor detoxifying S-RNase in *Prunus*. Acta Hortic.

[55] Lavin M, Herendeen PS, Wojciechowski MF (2005). Evolutionary rates analysis of Leguminosae implicates a rapid diversification of lineages during the tertiary. Syst Biol.

[56] Tsuchimatsu T, Suwabe K, Shimizu-Inatsugi R, Isokawa S, Pavlidis P, Städler T (2010). Evolution of self-compatibility in *Arabidopsis* by a mutation in the male specificity gene. Nature.

[57] Ngo BX, Wakana A, Kim JH, Mori T, Sakai K (2010). Estimation of self-incompatibility *S* genotypes of *Citrus cultivars* and plants based on controlled pollination with restricted number of pollen grains. J Fac Agric Kyushu Univ.

[58] Distefano G, Caruso M, La Malfa S, Gentile A, Tribulato E (2009). Histological and molecular analysis of pollen–pistil interaction in clementine. Plant Cell Rep.

[59] Roiz L, Goren R, Shoseyov O (1995). Stigmatic RNase in calamondin (*Citrus reticulata* var. austera x Fortunella sp.). Physiol Plantarum.

[60] H-x M, Y-h Q, da Silva JA T, Ye Z-x, Hu G-b (2011). Cloning and expression analysis of *S-RNase* homologous gene in *Citrus reticulata* Blanco cv. Wuzishatangju Plant Sci.

[61] Chai L, Ge X, Xu Q, Deng X (2011). *CgSL2*, an *S*-like RNase gene in ‘Zigui shatian’pummelo (*Citrus grandis* Osbeck), is involved in ovary senescence. Mol Biol Rep.

[62] Miao H-X, Qin Y-H, Ye Z-X, Hu G-B (2013). Molecular characterization and expression analysis of *ubiquitin-activating enzyme E1* gene in *Citrus reticulata*. Gene.

[63] Caruso M, Merelo P, Distefano G, La Malfa S, Piero ARL, Tadeo FR (2012). Comparative transcriptome analysis of stylar canal cells identifies novel candidate genes implicated in the self-incompatibility response of *Citrus clementina*. BMC Plant Biol.

[64] Ford CS, Wilkinson MJ (2012). Confocal observations of late-acting self-incompatibility in *Theobroma cacao* L. Sex Plant Reprod.

[65] Tate JA, Simpson BB (2004). Breeding system evolution in *Tarasa* (Malvaceae) and selection for reduced pollen grain size in the polyploid species. Am J Bot.

[66] Tao R, Watari A, Hanada T, Habu T, Yaegaki H, Yamaguchi M (2007). Self-compatible peach (*Prunus persica*) has mutant versions of the *S* haplotypes found in self-incompatible *Prunus* species. Plant Mol Biol.

[67] Broothaerts W, Janssens GA, Proost P, Broekaert WF (1995). cDNA cloning and molecular analysis of two self-incompatibility alleles from apple. Plant Mol Biol.

[68] Vieira J, Morales-Hojas R, Santos RA, Vieira CP (2007). Different positively selected sites at the gametophytic self-incompatibility pistil S-RNase gene in the Solanaceae and Rosaceae (*Prunus*, *Pyrus*, and *Malus*). J Mol Evol.

[69] Matsumoto D, Yamane H, Tao R (2008). Characterization of *SLFL1*, a pollen-expressed F-box gene located in the *Prunus S* locus. Sex Plant Reprod.

[70] Sassa H, Kakui H, Minamikawa M (2010). Pollen-expressed F-box gene family and mechanism of S-RNase-based gametophytic self-incompatibility (GSI) in Rosaceae. Sex Plant Reprod.

[71] Dhar R, Sharma N, Sharma B (2006). Ovule abortion in relation to breeding system in four *Trifolium* species. Curr Sci.

[72] Ištvánek J, Jaroš M, Křenek A, Řepková J (2014). Genome assembly and annotation for red clover (Trifolium pratense; Fabaceae). Am J Bot.

[73] Lawrence M (1996). Number of incompatibility alleles in clover and other species. Heredity.

[74] Vilanova S, Badenes ML, Burgos L, Martínez-Calvo J, Llácer G, Romero C (2006). Self-compatibility of two apricot selections is associated with two pollen-part mutations of different nature. Plant Physiol.

[75] Zuriaga E, Muñoz-Sanz JV, Molina L, Gisbert AD, Badenes ML, Romero C (2013). An *S*-Locus independent pollen factor confers self-compatibility in ‘Katy’ Apricot. PLoS One.

[76] Bechsgaard JS, Castric V, Charlesworth D, Vekemans X, Schierup MH (2006). The transition to self-compatibility in *Arabidopsis thaliana* and evolution within *S*-haplotypes over 10 Myr. Mol Biol Evol.

[77] Boggs NA, Nasrallah JB, Nasrallah ME (2009). Independent *S*-locus mutations caused self-fertility in *Arabidopsis thaliana*. PLoS Genet.

[78] Asmussen C (1993). Pollination biology of the sea pea, *Lathyrus japonicus*: floral characters and activity and flight patterns of bumblebees. Flora.

[79] López J, Rodríguez-Riaño T, Ortega-Olivencia A, Devesa JA, Ruiz T (1999). Pollination mechanisms and pollen-ovule ratios in some Genisteae (Fabaceae) from Southwestern Europe. Plant Syst Evol.

[80] Rodríguez-Riaño T (1997). Biología floral y reproductiva en Fabaceae de Extremadura.

[81] Rodet G, Vaissière BE, Brévault T, Grossa J-PT (1998). Status of self-pollen in bee pollination efficiency of white clover (*Trifolium repens* L.). Oecologia.

[82] Gibbs P, Sassaki R (1998). Reproductive biology of *Dalbergia miscolobium* Benth. (Leguminosae-Papilionoideae) in SE Brazil: the effects of pistillate sorting on fruit-set. Ann Bot.

[83] Rodríguez-Riaño T, Ortega-Olivencia A, Devesa JA (1964). Reproductive biology in *Cytisus multiflorus* (Fabaceae). Annales Botanici Fennici: 2004.

[84] Cooper D, Brink R (1940). Somatoplastic sterility as a cause of seed failure after interspecific hybridization. Genetics.

[85] Miri R, Bubar J (1966). Self-incompatibility as an outcrossing mechanism in birdsfoot trefoil (*Lotus corniculatus*). Can J Plant Sci.

[86] Seavey SR, Bawa KS (1986). Late-acting self-incompatibility in Angiosperms. Bot Rev.

[87] Valtueña FJ, Rodríguez-Riaño T, Espinosa F, Ortega-Olivencia A (2010). Self-sterility in two *Cytisus* species (Leguminosae, Papilionoideae) due to early-acting inbreeding depression. Am J Bot.

[88] Waser NM, Price MV (1991). Reproductive costs of self-pollination in *Ipomopsis aggregata* (Polemoniaceae) - are ovules usurped?. Am J Bot.

[89] Gibbs PE, Bianchi M (1993). Postpollination events in species of *Chorisia* (Bombacaceae) and *Tabebuia* (Bignoniaceae) with late-acting self-incompatibility. Bot Acta.

[90] Rice P, Longden I, Bleasby A (2000). EMBOSS: the European molecular biology open software suite. Trends Genet.

[91] Johnson M, Zaretskaya I, Raytselis Y, Merezhuk Y, McGinnis S, Madden TL (2008). NCBI BLAST: a better web interface. Nucleic Acids Res.

[92] Young ND, Debellé F, Oldroyd GE, Geurts R, Cannon SB, Udvardi MK (2011). The *Medicago* genome provides insight into the evolution of rhizobial symbioses. Nature.

[93] Varshney RK, Song C, Saxena RK, Azam S, Yu S, Sharpe AG (2013). Draft genome sequence of chickpea (*Cicer arietinum*) provides a resource for trait improvement. Nat Biotech.

[94] Schmutz J, Cannon SB, Schlueter J, Ma J, Mitros T, Nelson W (2010). Genome sequence of the palaeopolyploid soybean. Nature.

[95] Yang H, Tao Y, Zheng Z, Shao D, Li Z, Sweetingham MW (2013). Rapid development of molecular markers by next-generation sequencing linked to a gene conferring phomopsis stem blight disease resistance for marker-assisted selection in lupin (*Lupinus angustifolius* L.) breeding. Theor Appl Genet.

[96] Artimo P, Jonnalagedda M, Arnold K, Baratin D, Csardi G, de Castro E (2012). ExPASy: SIB bioinformatics resource portal. Nucleic Acids Res.

[97] Reboiro-Jato D, Reboiro-Jato M, Fdez-Riverola F, Vieira CP, Fonseca NA, Vieira J (2012). ADOPS - Automatic Detection Of Positively Selected Sites. J Integr Bioinform.

[98] Huelsenbeck JP, Ronquist F (2001). MRBAYES: Bayesian inference of phylogenetic trees. Bioinformatics.

[99] Tamura K, Peterson D, Peterson N, Stecher G, Nei M, Kumar S (2011). MEGA5: molecular evolutionary genetics analysis using maximum likelihood, evolutionary distance, and maximum parsimony methods. Mol Biol Evol.

[100] Ingram GC, Doyle S, Carpenter R, Schultz EA, Simon R, Coen ES (1997). Dual role for fimbriata in regulating floral homeotic genes and cell division in *Antirrhinum*. Embo J.

[101] Rozas J, Sanchez-DelBarrio JC, Messeguer X, Rozas R (2003). DnaSP, DNA polymorphism analyses by the coalescent and other methods. Bioinformatics.

[102] Benedito VA, Torres‐Jerez I, Murray JD, Andriankaja A, Allen S, Kakar K (2008). A gene expression atlas of the model legume *Medicago truncatula*. The Plant J.

[103] Rodríguez-Riaño T, Valtueña FJ, Ortega-Olivencia A (2006). Megasporogenesis, megagametogenesis and ontogeny of the aril in *Cytisus striatus* and *C. multiflorus* (Leguminosae: Papilionoideae). Ann Bot.

[104] Blankenberg D, Kuster GV, Coraor N, Ananda G, Lazarus R, Mangan M (2010). Galaxy: a web‐based genome analysis tool for experimentalists. Current protocols in molecular biology.

[105] Giardine B, Riemer C, Hardison RC, Burhans R, Elnitski L, Shah P (2005). Galaxy: a platform for interactive large-scale genome analysis. Genome Res.

[106] Goecks J, Nekrutenko A, Taylor J (2010). Galaxy: a comprehensive approach for supporting accessible, reproducible, and transparent computational research in the life sciences. Genome Biol.

[107] Haas BJ, Papanicolaou A, Yassour M, Grabherr M, Blood PD, Bowden J (2013). De novo transcript sequence reconstruction from RNA-seq using the Trinity platform for reference generation and analysis. Nat Protoc.

[108] Roberts A, Pachter L (2013). Streaming fragment assignment for real-time analysis of sequencing experiments. Nat Methods.

[109] Conesa A, Götz S, García-Gómez JM, Terol J, Talón M, Robles M (2005). Blast2GO: a universal tool for annotation, visualization and analysis in functional genomics research. Bioinformatics.

