# Supplementary figures and images for: No evidence for Fabaceae Gametophytic self-incompatibility being determined by Rosaceae, Solanaceae, and Plantaginaceae S-RNase lineage genes
Source: BMC Plant Biol. 2015 Jun 2;15:129. doi: 10.1186/s12870-015-0497-2 (PMC4451870; doi:10.1186/s12870-015-0497-2)

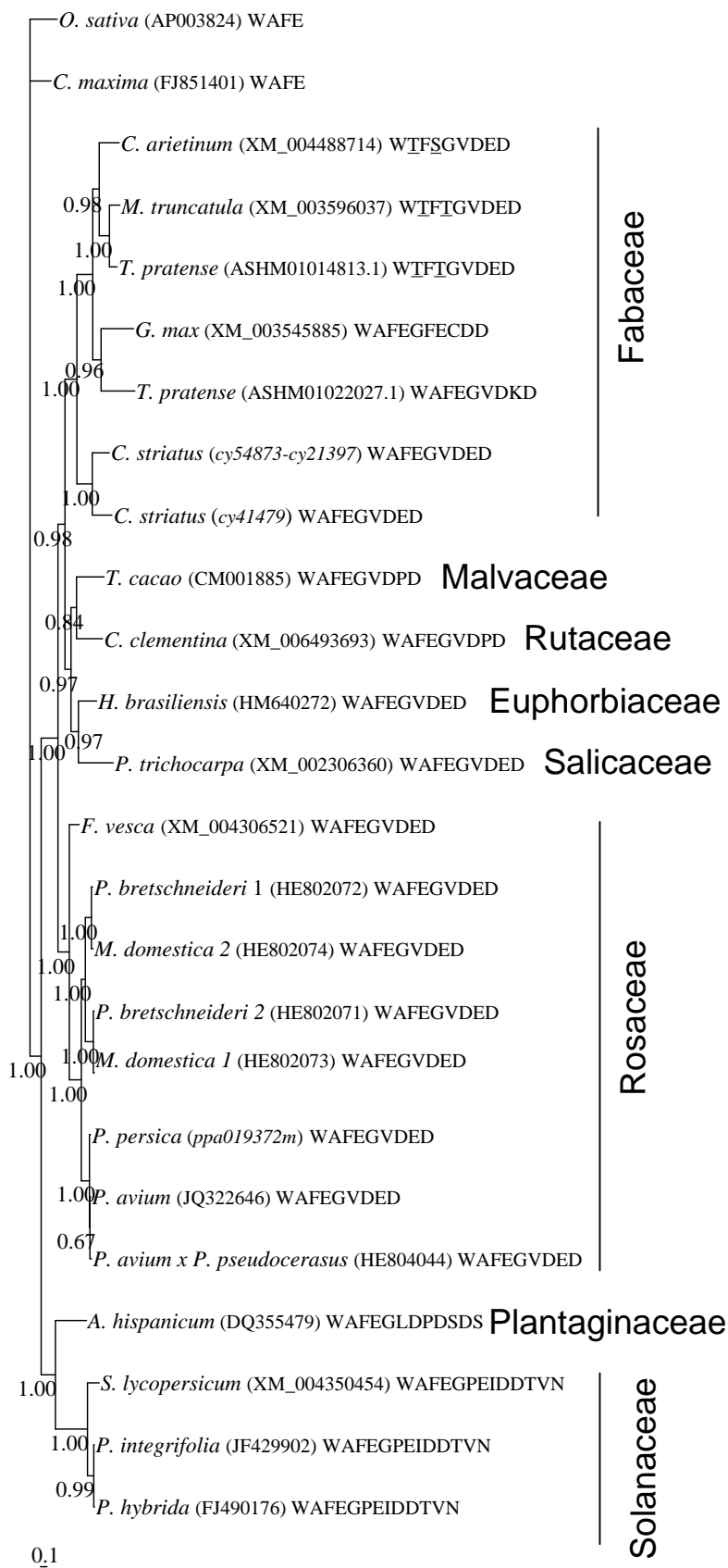

A

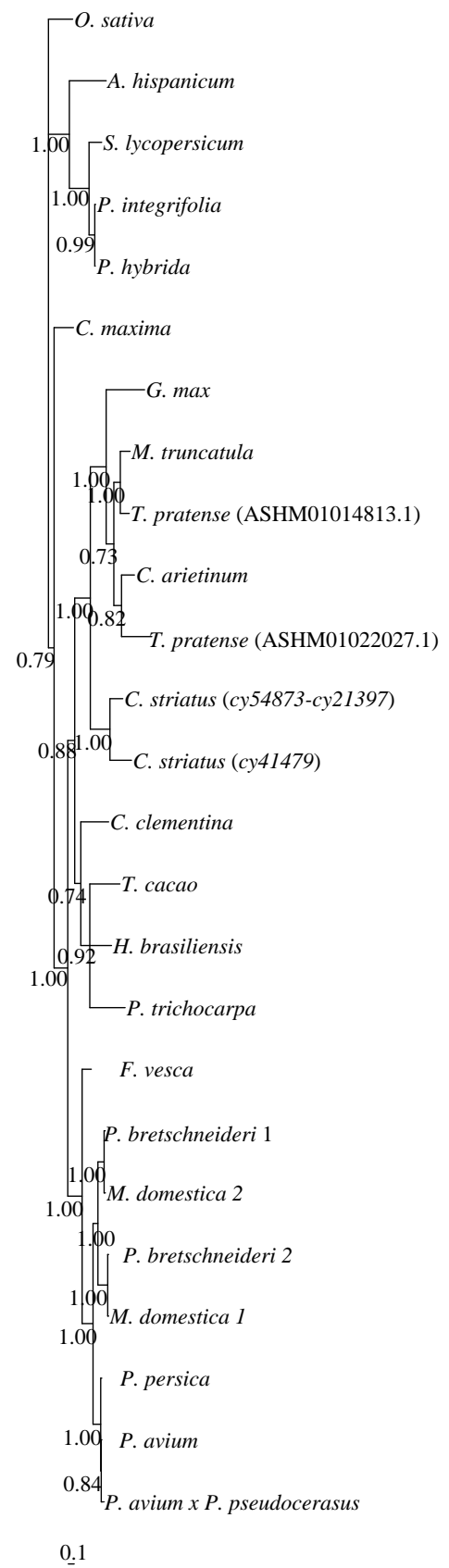

B

Supplement: Additional file 1: — Bayesian phylogenetic trees showing the relationship of SSK1 like genes in flowering plants. Sequences were aligned using ClustalW2 (A), and T-coffee (B) algorithms. The tree was rooted using O. sativa ([GenBank:AP003824]) and C. maxima ([GenBank:FJ851401]) genes. Numbers below the branches represent posterior credibility values above 60. [file 12870_2015_497_MOESM1_ESM.pdf]

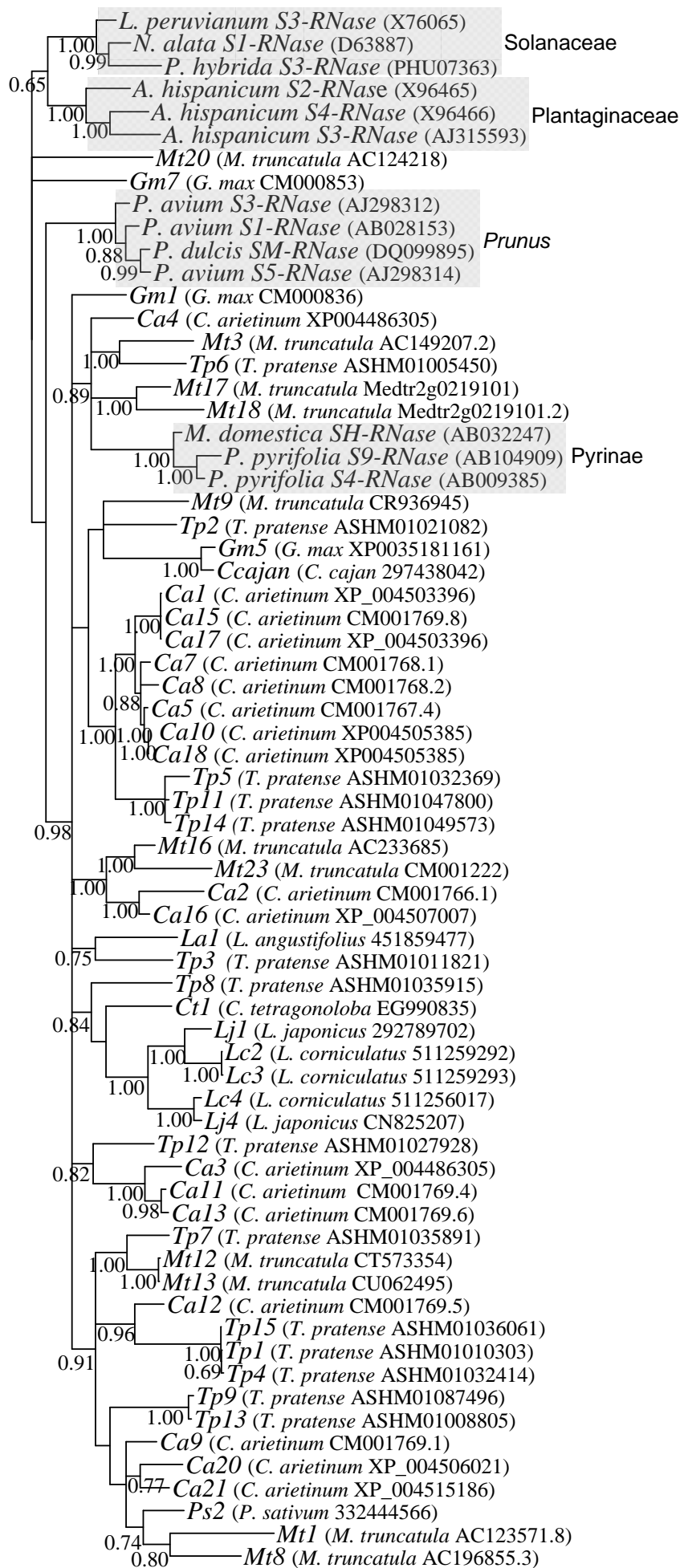

A

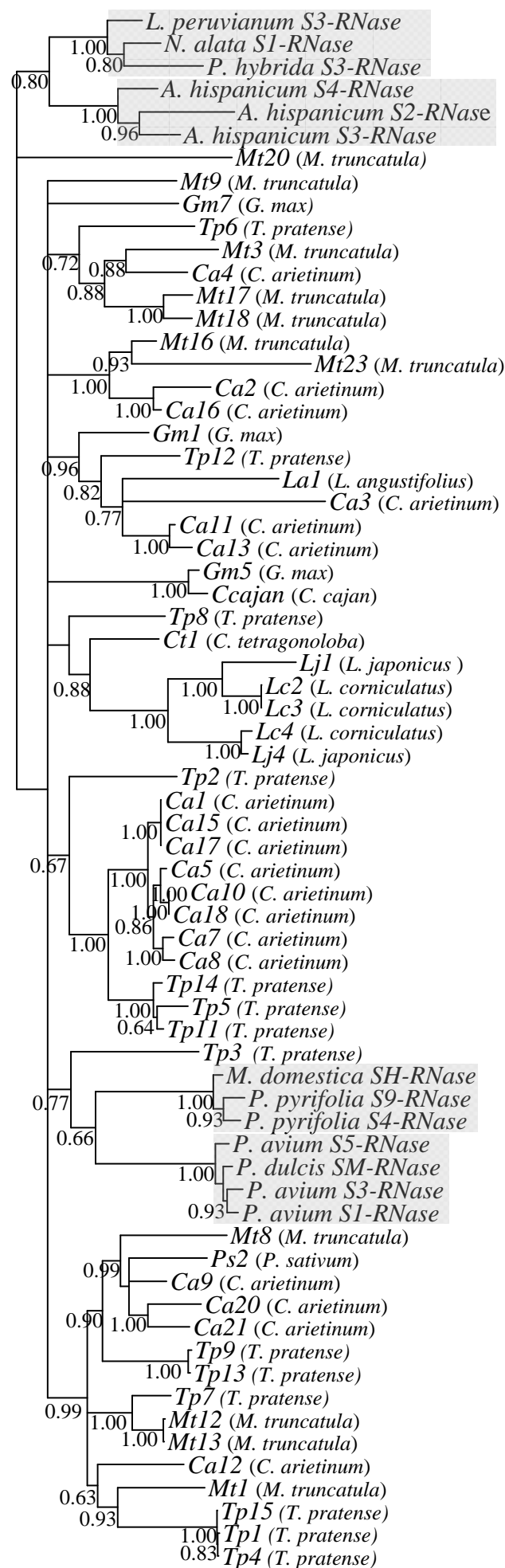

B

Supplement: Additional file 2: — Bayesian phylogenetic trees showing the relationship of Fabaceae S-RNase lineage genes and Prunus , Pyrinae, Solanaceae and Plantaginaceae S-RNases. Sequences were aligned using ClustalW2 (A), and T-coffee (B) algorithms. The reference sequences are shaded. [file 12870_2015_497_MOESM2_ESM.pdf]

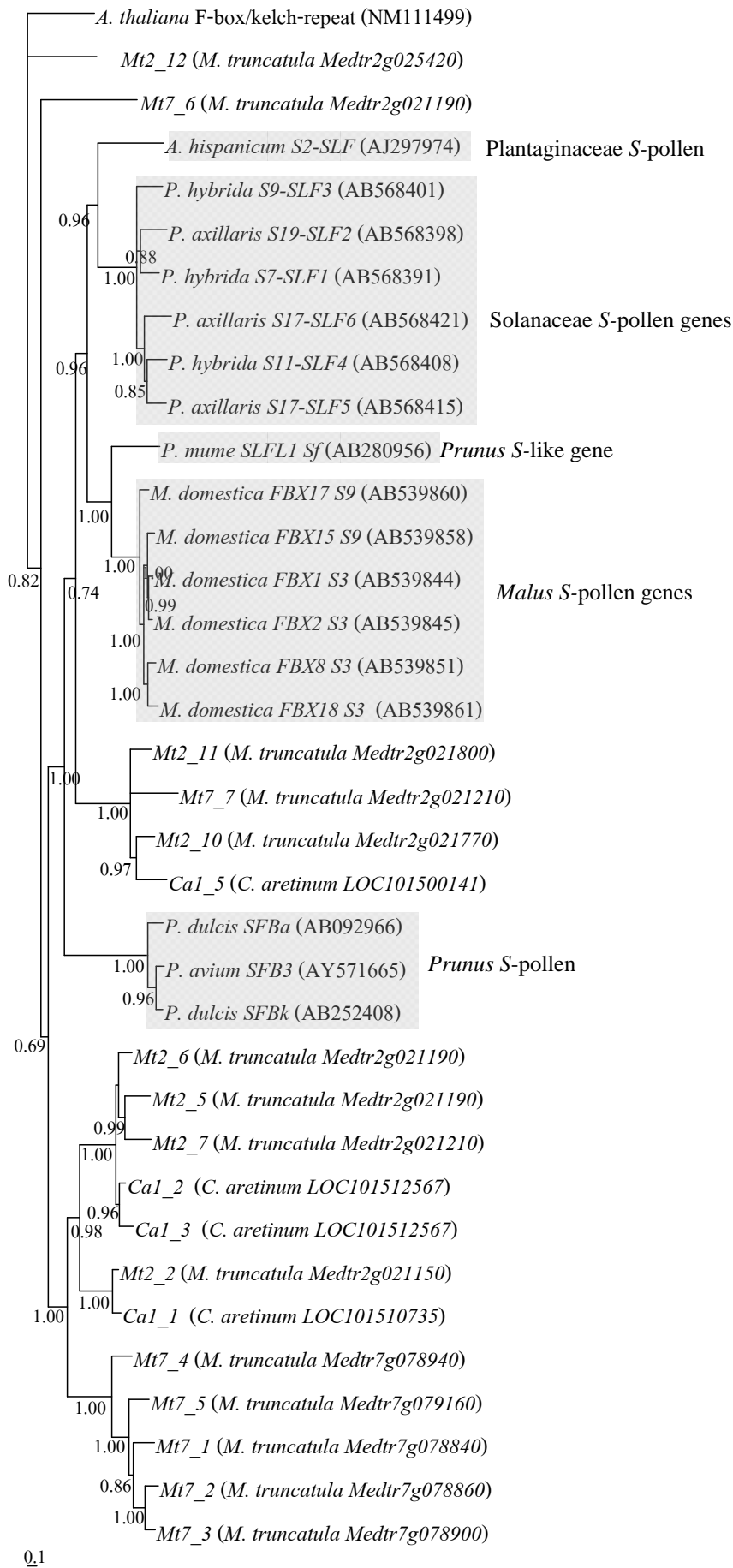

A

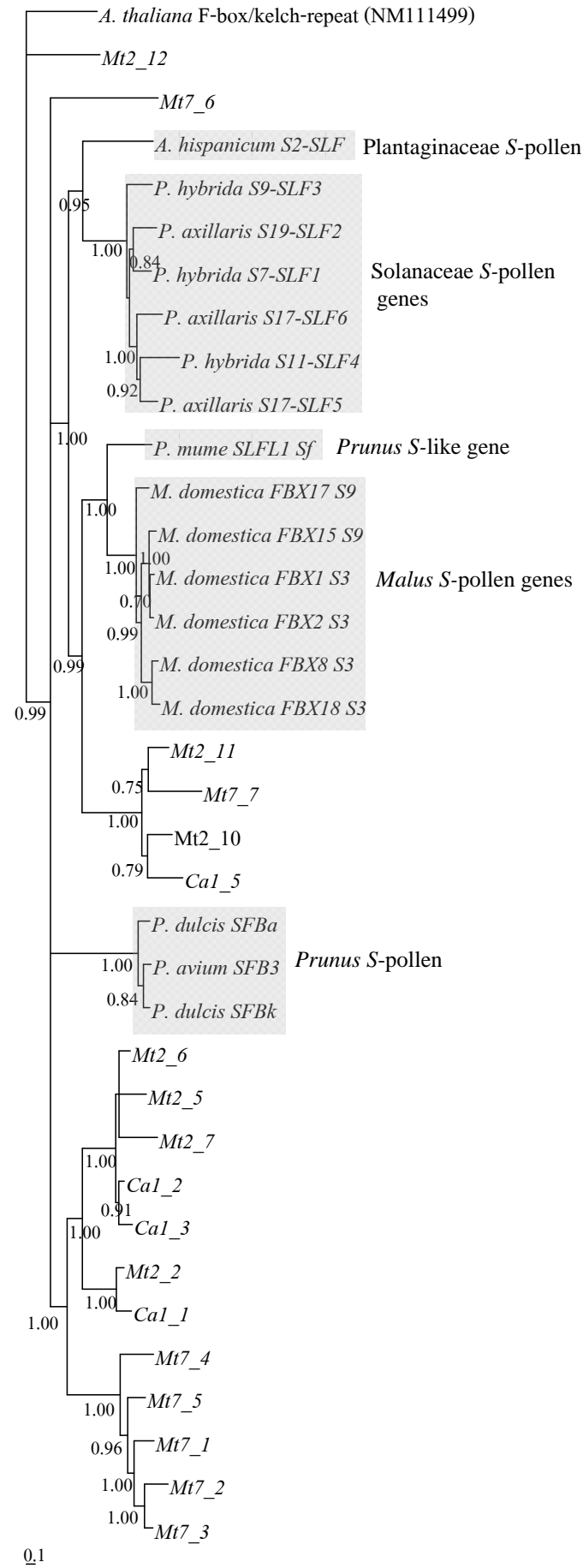

B

Supplement: Additional file 5: — Bayesian phylogenetic trees showing the relationship of the F-box SFB - SFBB - and SLFL - like genes surrounding the C. arietinum Ca4 , M. truncatula Mt3 , Mt17 , Mt18 , and Mt20 genes, and S- pollen genes from Prunus , Malus , Solanaceae and Plantaginaceae, and Prunus S -like genes (shaded sequences). Sequences were aligned using ClustalW2 (A), and T-coffee (B) algorithms. The tree was rooted using A. thaliana F-box/kelch-repeat ([GenBank:NM111499]) gene. Numbers below the branches represent posterior credibility values above 60. [file 12870_2015_497_MOESM5_ESM.pdf]

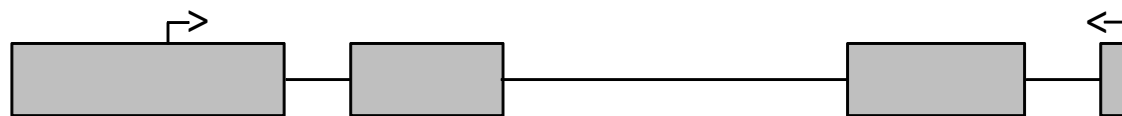

A

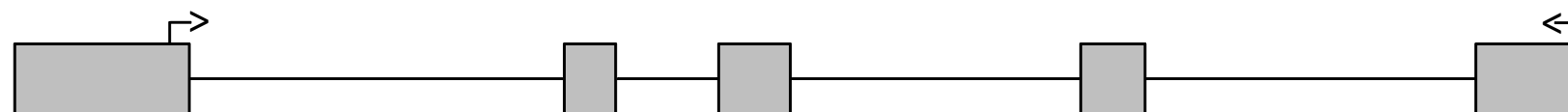

B

100 bp

**Additional file 7**

Supplement: Additional file 7: — The 100 most expressed genes of the C. striatus stigma with style transcriptome. [file 12870_2015_497_MOESM7_ESM.pdf]
