# Supplementary material for: No evidence for Fabaceae Gametophytic self-incompatibility being determined by Rosaceae, Solanaceae, and Plantaginaceae S-RNase lineage genes
Source: BMC Plant Biol. 2015 Jun 2;15:129. doi: 10.1186/s12870-015-0497-2 (PMC4451870; doi:10.1186/s12870-015-0497-2)
Supplement: Additional file 3: — Fabaceae T2-RNases available in GenBank not presenting amino acid pattern 4. [file 12870_2015_497_MOESM3_ESM.pdf]

Additional file 3. Fabaceae *T2-RNases* available in GenBank, not presenting in their amino acid sequence amino acid pattern 4 [26]

Gene name	Gene code	PI	Motif 1	Motif 2	Motif 4
Lotus corniculatus					
511246152	*Lc1*	5.54	FTIHGLWPDY	WPSLSCG	
511259292	*Lc2*	8.66	FTIHGLWPST	QPNWCQP	-
511259293	*Lc3*	8.78	FTIHGLWPST	WPDMIKPT	-
511256017	*Lc4*	9.36	FTIHGLWPGN	WPNMLKA	-
L. japonicus					
292789702 (AK339271)	*Lj1*	9.78	LVIHGLWPSN	WPEMLPP	-
BT148442	*Lj2*	7.55	FTIHGLWPGN	WPNMLAT	-
388505137 BT140840	*Lj3*	6.79	FTIHGLWPGN	WPNMLAA	-
CN825207 (AFK37221)	*Lj4*	6.34	FTIHGLWPGN	WPNMLAA	-
Arachis hypogaea					
298122009	*Ah1*	5.14	FTIHGLWTDY	WPSYSCS	-
372398525	*Ah2*	6.24	FTIHGLWPTN	WPSLQPG	-
Pisum sativum					
332432295	*Ps1*	5.94	FVIHGLWPSK	WPALFEN	-
332444566	*Ps2*	8.82	FTIHGLWPSK	WPALFKK	-
Cajanus cajan					
297438042	*Ccajan*	8.59	FTIYYLRPRK	WPDLRTD	
Lens culinaris					

331110541	*Lens1*	6.16	FTIHGLWPDY	WPSLSCG	SPSSCS
Cyamopsis tetragonoloba					
EG990835	*Ct1*	8,86	FTIHGLWPAN	WPNLKGT	-
Medicago sativa					
335964967	*Ns1*	5.69	FTIHGLWPDY	WPSLSCG	SPSSCS

Underscored are amino acids that are not allowed in the motifs of [26]
