# Supplementary material for: No evidence for Fabaceae Gametophytic self-incompatibility being determined by Rosaceae, Solanaceae, and Plantaginaceae S-RNase lineage genes
Source: BMC Plant Biol. 2015 Jun 2;15:129. doi: 10.1186/s12870-015-0497-2 (PMC4451870; doi:10.1186/s12870-015-0497-2)
Supplement: Additional file 4: — Reads from the SRP033257 experiment of M. truncatula (RNA-Seq data sets from a mixed sample of M. truncatula root knot galls infected with Meloidogyne hapla (a plant-nematode)) supporting the expression of the Mt3 , Mt17 , Mt18 , and Mt_10 genes. [file 12870_2015_497_MOESM4_ESM.pdf]

Additional file 4: Reads supporting expression of the *Mt3*, *Mt17*, *Mt18*, and *Mt2_10* genes in SRP033257 experiment of *M. truncatula* (RNA-Seq data from a mixed sample of *M. truncatula* root knot galls infected with *Meloidogyne hapla* (a plant-nematode))

Gene	Reads
Mt3	gnl|SRA|SRR1043883.43136986.1
	gnl|SRA|SRR1043883.32010848.1
	gnl|SRA|SRR1043797.10726404.1
	gnl|SRA|SRR1043795.25285202.1
	gnl|SRA|SRR1043779.54087688.1
	gnl|SRA|SRR1043666.57815839.1
Mt17	gnl|SRA|SRR1043885.10658370.1
	gnl|SRA|SRR1043881.2087054.1
	gnl|SRA|SRR1043808.4289521.1
	gnl|SRA|SRR1043798.35907529.1
	gnl|SRA|SRR1043784.24879450.1
	gnl|SRA|SRR1043742.55576161.1
	gnl|SRA|SRR1043684.56906268.1
	gnl|SRA|SRR1043605.31305766.1
	gnl|SRA|SRR1043107.12591822.1
	gnl|SRA|SRR1043068.51016104.1
Mt18	gnl|SRA|SRR1043874.15040445.1
	gnl|SRA|SRR1043781.31064223.1
	gnl|SRA|SRR1043778.2254378.1
	gnl|SRA|SRR1043106.15306711.1
Mt2_10	gnl|SRA|SRR1043886.27606493.1
	gnl|SRA|SRR1043876.38060156.1
	gnl|SRA|SRR1043744.3162043.1

	gnl|SRA|SRR1043713.23430899.1
	gnl|SRA|SRR1043686.23125586.1
	gnl|SRA|SRR1043674.18978417.1
	gnl|SRA|SRR1043106.35104704.1
