# Supplementary material for: No evidence for Fabaceae Gametophytic self-incompatibility being determined by Rosaceae, Solanaceae, and Plantaginaceae S-RNase lineage genes
Source: BMC Plant Biol. 2015 Jun 2;15:129. doi: 10.1186/s12870-015-0497-2 (PMC4451870; doi:10.1186/s12870-015-0497-2)
Supplement: Additional file 6: — Bayesian phylogenetic trees, showing the relationship of the C. striatus CsRNase1 and CsRNase2 genes with Fabaceae S-RNase lineage genes and Prunus , Pyrinae, Solanaceae and Plantaginaceae S-RNases (shaded sequences). Sequences were aligned using ClustalW2 (A), and T-coffee (B) algorithms. Numbers below the branches represent posterior credibility values above 60. [file 12870_2015_497_MOESM6_ESM.pdf]

**Additional file** 6. The 100 most expressed genes of the *C. striatus* stigma with style transcriptome

| Gene | FPKM | Longest isoform | Length (bp) | Sequence Description | InterProScan (PFAM) | GO terms | Mean similarity (%) |
|---|---|---|---|---|---|---|---|
| *c48578_g1* | 15380.77 | *c48578_g1_i1* | 804 | NA | IPR006501 | F:pectinesterase activity; F:enzyme inhibitor activity | NA |
| *c50936_g1* | 12242.09 | *c50936_g1_i9* | 997 | proline-rich protein 4-like | no IPS match | NA | NA |
| *c52888_g1* | 10478.76 | *c52888_g1_i6* | 1921 | l-ascorbate oxidase-like | IPR011707; IPR011706; IPR001117 | F:copper ion binding; F:oxidoreductase activity; P:oxidation-reduction process | 72.24 |
| *c51372_g2* | 9746.66 | *c51372_g2_i1* | 1161 | 1-aminocyclopropane-1-carboxylate oxidase | IPR005123; IPR026992 | P:oxidation-reduction process; F:oxidoreductase activity | 92.40 |
| *c19588_g1* | 9687.56 | *c19588_g1_i1* | 208 | NA | no IPS match | NA | NA |
| *c48712_g1* | 8535.12 | *c48712_g1_i2* | 773 | pollen ole e 1 allergen and extensin family | no IPS match | NA | 64.65 |
| *c51468_g2* | 7286.2 | *c51468_g2_i1* | 1050 | lipid transfer | IPR016140 | NA | 73.30 |
| *c48699_g2* | 7201.71 | *c48699_g2_i2* | 1035 | fasciclin-like arabinogalactan protein 3-like | no IPS match | NA | 71.55 |
| *c52392_g6* | 7038.02 | *c52392_g6_i1* | 365 | pectinesterase inhibitor domain | IPR000070 | C:cell wall; P:cell wall modification; F:pectinesterase activity | 66.55 |
| *c90467_g1* | 6788.62 | *c90467_g1_i1* | 569 | NA | no IPS match | NA | NA |
| *c50936_g2* | 6738.06 | *c50936_g2_i1* | 177 | repetitive proline-rich cell wall protein 1-like | no IPS match | NA | 66.56 |
| *c50110_g2* | 6484.17 | *c50110_g2_i1* | 2534 | tonoplast intrinsic | no IPS match | NA | 91.70 |
| *c46921_g3* | 6476.23 | *c46921_g3_i1* | 2136 | senescence-associated | no IPS match | NA | 88.90 |
| *c48255_g4* | 6332.68 | *c48255_g4_i1* | 350 | NA | no IPS match | NA | NA |
| *c16527_g1* | 5646.63 | *c16527_g1_i1* | 178 | NA | no IPS match | NA | NA |
| *c45810_g2* | 5446.95 | *c45810_g2_i3* | 799 | NA | no IPS match | NA | NA |
| *c47271_g2* | 4948.52 | *c47271_g2_i3* | 833 | plant invertase pectin methylesterase inhibitor | IPR006501 | F:pectinesterase activity; F:enzyme inhibitor activity | 58.40 |
| *c47242_g1* | 4940.39 | *c47242_g1_i1* | 670 | NA | no IPS match | NA | NA |
| *c48578_g2* | 4883.16 | *c48578_g2_i1* | 1020 | hypothetical ENH_00061390 | no IPS match | NA | 78.00 |

| | | | | | | | |
|---|---|---|---|---|---|---|---|
| *c45963_g2* | 4459.43 | *c45963_g2_i3* | 1085 | kda proline-rich | IPR027923 | NA | 89.55 |
| *c43040_g1* | 4441.74 | *c43040_g1_i1* | 695 | NA | no IPS match | NA | NA |
| *c42721_g1* | 4312.93 | *c42721_g1_i2* | 338 | cold dehydrin | no IPS match | NA | 74.25 |
| *c46311_g1* | 4214.36 | *c46311_g1_i1* | 1705 | s-like ribonuclease | IPR001568 | F:ribonuclease T2 activity; F:RNA binding | 48.80 |
| *c42336_g1* | 4193.27 | *c42336_g1_i1* | 944 | invertase inhibitor-like | IPR006501 | F:enzyme inhibitor activity; F:pectinesterase activity | 73.40 |
| *c46687_g1* | 4079.14 | *c46687_g1_i2* | 894 | uncharacterized LOC100305719 | IPR003496 | P:response to stress | 74.00 |
| *c75788_g1* | 4034.93 | *c75788_g1_i1* | 1025 | p21 family | IPR001938 | NA | 87.05 |
| *c48812_g1* | 3930.24 | *c48812_g1_i3* | 1260 | NA | no IPS match | NA | NA |
| *c51659_g1* | 3779.05 | *c51659_g1_i1* | 590 | NA | no IPS match | NA | NA |
| *c50501_g2* | 3754.39 | *c50501_g2_i2* | 2312 | pectinesterase inhibitor-like | IPR000070; IPR006501 | F:pectinesterase activity; P:cell wall modification; C:cell wall; F:enzyme inhibitor activity | 74.15 |
| *c90364_g1* | 3627.86 | *c90364_g1_i1* | 517 | NA | no IPS match | NA | NA |
| *c45961_g1* | 3489.33 | *c45961_g1_i1* | 667 | NA | no IPS match | NA | NA |
| *c52061_g1* | 3421.89 | *c52061_g1_i2* | 1057 | pectinesterase inhibitor 28-like | IPR000070 | C:cell wall; P:cell wall modification; F:pectinesterase activity | 84.85 |
| *c22143_g1* | 3230.07 | *c22143_g1_i1* | 865 | mlp-like protein 28 | IPR000916 | P:defense response; P:response to biotic stimulus | 68.60 |
| *c75794_g1* | 3125.87 | *c75794_g1_i1* | 680 | NA | no IPS match | NA | NA |
| *c51219_g2* | 2960.16 | *c51219_g2_i2* | 1617 | s-adenosylmethionine decarboxylase | no IPS match | NA | 85.95 |
| *c50488_g7* | 2936.95 | *c50488_g7_i1* | 1269 | major intrinsic protein family transporter | IPR000425 | P:transport; C:membrane; F:transporter activity | 94.25 |
| *c48144_g1* | 2933.95 | *c48144_g1_i7* | 733 | ubiquitin-like | no IPS match | NA | 99.50 |
| *c40350_g1* | 2790.6 | *c40350_g1_i1* | 275 | NA | no IPS match | NA | NA |
| *c52392_g5* | 2773.69 | *c52392_g5_i1* | 1056 | pectinesterase inhibitor | IPR000070 | P:cell wall modification; C:cell wall; F:pectinesterase activity | 71.15 |
| *c51003_g1* | 2753.36 | *c51003_g1_i1* | 729 | xyloglucan endotransglucosylase | IPR010713 | P:cellular glucan metabolic process; F:xyloglucan:xyloglucosyl transferase activity; C:cell wall; C:apoplast | 92.90 |
| *c51003_g2* | 2654.02 | *c51003_g2_i1* | 617 | endo-xyloglucan transferase | IPR000757 | P:carbohydrate metabolic process; F:hydrolase activity, hydrolyzing O-glycosyl compounds | 94.40 |

| | | | | | | | |
|---|---|---|---|---|---|---|---|
| *c50255_g2* | 2653.13 | *c50255_g2_i1* | 315 | ubiquitin-like partial | IPR000626 | F:protein binding | 99.50 |
| *c42538_g1* | 2644.45 | *c42538_g1_i1* | 809 | dehydrin b | IPR000167 | P:response to stress; P:response to water stimulus | 55.55 |
| *c46921_g1* | 2601.19 | *c46921_g1_i1* | 1781 | senescence-associated partial | no IPS match | NA | 83.55 |
| *c46492_g1* | 2463.08 | *c46492_g1_i1* | 856 | 21 kda protein | no IPS match | NA | 84.45 |
| *c46038_g1* | 2437.19 | *c46038_g1_i3* | 467 | late embryogenesis abundant protein family | no IPS match | NA | 87.50 |
| *c46271_g2* | 2429.47 | *c46271_g2_i1* | 375 | cytochrome b-c1 complex subunit 8-like | IPR020101 | C:respiratory chain; C:mitochondrial inner membrane; P:electron transport chain | 85.80 |
| *c52202_g1* | 2416.04 | *c52202_g1_i1* | 609 | pectate lyase-like | IPR002022 | - | 83.50 |
| *c49613_g1* | 2376.49 | *c49613_g1_i2* | 1570 | polygalacturonase-like | IPR000743 | P:carbohydrate metabolic process; F:polygalacturonase activity | 80.80 |
| *c44507_g1* | 2363.77 | *c44507_g1_i2* | 598 | b12d protein | IPR010530 | NA | 89.30 |
| *c52982_g1* | 2355.48 | *c52982_g1_i1* | 2274 | atpase plasma membrane-type | IPR008250; IPR023214 | F:nucleotide binding; F:metal ion binding | 93.15 |
| *c1249_g1* | 2352.19 | *c1249_g1_i1* | 485 | cell wall | no IPS match | NA | 70.17 |
| *c35223_g1* | 2349.05 | *c35223_g1_i1* | 1392 | gdsl esterase lipase at5g33370-like | IPR001087 | P:lipid metabolic process; F:hydrolase activity, acting on ester bonds | 88.20 |
| *c39168_g1* | 2346.25 | *c39168_g1_i1* | 230 | hypothetical PHAVU_011G150500g | no IPS match | NA | 88.00 |
| *c48584_g3* | 2327.12 | *c48584_g3_i1* | 443 | protein ralf-like 9-like | IPR008801 | NA | 58.25 |
| *c50298_g1* | 2302.94 | *c50298_g1_i3* | 515 | pectinesterase ppme1 | no IPS match | NA | 76.05 |
| *c47149_g2* | 2266.48 | *c47149_g2_i2* | 905 | lipid transfer protein precursor | no IPS match | NA | 75.80 |
| *c47764_g3* | 2184.35 | *c47764_g3_i1* | 652 | acid beta-fructofuranosidase | IPR013189 | NA | 92.70 |
| *c44734_g1* | 2184.03 | *c44734_g1_i3* | 3141 | cell wall-associated hydrolase | no IPS match | NA | 82.00 |
| *c41993_g1* | 2152.52 | *c41993_g1_i1* | 790 | cell wall | no IPS match | NA | 61.28 |
| *c52221_g1* | 2147.31 | *c52221_g1_i4* | 679 | NA | no IPS match | NA | |
| *c39797_g1* | 2137.55 | *c39797_g1_i1* | 868 | mlp-like protein 423-like | IPR000916 | P:response to biotic stimulus; P:defense response | 83.55 |
| *c46440_g2* | 2129.59 | *c46440_g2_i1* | 471 | arabinogalactan peptide 16-like | no IPS match | NA | 83.39 |
| *c46355_g1* | 2075.15 | *c46355_g1_i1* | 785 | NA | IPR015157 | NA | NA |

| | | | | | | | |
|---|---|---|---|---|---|---|---|
| *c49376_g2* | 2039.82 | *c49376_g2_i2* | 492 | late embryogenesis abundant protein 1-like | no IPS match | - | 77.45 |
| *c52202_g2* | 2013.73 | *c52202_g2_i2* | 943 | pectate lyase-like | IPR007524; IPR002022 | F:pectate lyase activity | 75.85 |
| *c49407_g1* | 2006.4 | *c49407_g1_i1* | 974 | stem 28 kda glycoprotein | no IPS match | NA | 82.00 |
| *c46921_g2* | 2002.22 | *c46921_g2_i1* | 2594 | rrna intron-encoded homing endonuclease | no IPS match | NA | 76.80 |
| *c31965_g1* | 1962.53 | *c31965_g1_i1* | 790 | ekn protein | no IPS match | NA | 66.00 |
| *c45615_g2* | 1943.6 | *c45615_g2_i3* | 883 | ribulose- -bisphosphate carboxylase oxygenase small subunit | IPR000894; IPR024680 | NA | 90.05 |
| *c48699_g1* | 1942.53 | *c48699_g1_i1* | 623 | NA | no IPS match | NA | |
| *c40016_g1* | 1932.13 | *c40016_g1_i1* | 808 | non-specific lipid-transfer protein 2-like | IPR016140 | NA | 86.00 |
| *c35211_g1* | 1925.86 | *c35211_g1_i1* | 738 | leguminosin group485 secreted peptide | no IPS match | NA | 64.75 |
| *c51694_g1* | 1922.15 | *c51694_g1_i4* | 1636 | pectinesterase pectinesterase inhibitor | IPR000070; IPR006501 | F:pectinesterase activity; C:cell wall; P:cell wall modification; F:enzyme inhibitor activity | 72.85 |
| *c51082_g2* | 1885.87 | *c51082_g2_i3* | 2125 | pectinesterase inhibitor 45-like | IPR006501; IPR000070 | F:pectinesterase activity; F:enzyme inhibitor activity; P:cell wall modification; C:cell wall | 73.85 |
| *c52392_g2* | 1880.4 | *c52392_g2_i1* | 762 | pectinesterase inhibitor 21-like | IPR006501 | F:enzyme inhibitor activity; F:pectinesterase activity | 72.60 |
| *c49201_g4* | 1877.53 | *c49201_g4_i2* | 325 | calmodulin | IPR002048 | F:calcium ion binding | 100.00 |
| *c52376_g4* | 1869.32 | *c52376_g4_i3* | 1855 | alpha tubulin 1 | no IPS match | NA | 99.00 |
| *c52177_g1* | 1868.51 | *c52177_g1_i1* | 2100 | sucrose transport | no IPS match | NA | 79.90 |
| *c48584_g2* | 1846.64 | *c48584_g2_i1* | 1344 | microtubule organization 1 family | IPR008801 | NA | 82.10 |
| *c41972_g2* | 1777.82 | *c41972_g2_i1* | 444 | probable pectate lyase 3-like | no IPS match | NA | 79.40 |
| *c51841_g1* | 1712.04 | *c51841_g1_i1* | 2865 | beta-galactosidase 13-like | IPR001944; IPR000922; IPR006104 | P:carbohydrate metabolic process; F:hydrolase activity, hydrolyzing O-glycosyl compounds; F:carbohydrate binding | 82.65 |
| *c47409_g1* | 1711.14 | *c47409_g1_i3* | 900 | protein ralf-like 19-like | IPR008801 | NA | 74.35 |
| *c33197_g1* | 1703.12 | *c33197_g1_i1* | 1118 | phosphate-responsive 1 family | IPR006766 | NA | 62.55 |

| | | | | | | | |
|---|---|---|---|---|---|---|---|
| *c35308_g1* | 1693.61 | *c35308_g1_i1* | 199 | NA | no IPS match | NA | NA |
| *c50150_g2* | 1658.2 | *c50150_g2_i1* | 1506 | Lipoxygenase | IPR013819 | F:metal ion binding; P:oxidation-reduction process; F:oxidoreductase activity | 88.15 |
| *c20413_g1* | 1627.37 | *c20413_g1_i1* | 386 | NA | no IPS match | NA | NA |
| *c51808_g2* | 1625.43 | *c51808_g2_i2* | 218 | sphingolipid delta -desaturase des1-like | no IPS match | NA | 95.90 |
| *c76225_g1* | 1624.68 | *c76225_g1_i1* | 686 | cysteine proteinase inhibitor | no IPS match | NA | 88.85 |
| *c51857_g1* | 1621.74 | *c51857_g1_i1* | 893 | adp-ribosylation factor 2-like | no IPS match | NA | 98.60 |
| *c50488_g3* | 1617.34 | *c50488_g3_i1* | 180 | aquaporin protein 7 | no IPS match | NA | 93.00 |
| *c48268_g1* | 1605.12 | *c48268_g1_i1* | 522 | polcalcin nic t 1-like | no IPS match | NA | 92.90 |
| *c49048_g1* | 1578.23 | *c49048_g1_i1* | 824 | NA | no IPS match | NA | NA |
| *c42408_g1* | 1556.53 | *c42408_g1_i1* | 505 | arabinogalactan peptide 23-like | no IPS match | NA | 66.33 |
| *c44616_g1* | 1555.27 | *c44616_g1_i1* | 882 | linoleate 9s-lipoxygenase-like | IPR013819 | F:metal ion binding; F:oxidoreductase activity; P:oxidation-reduction process | 89.25 |
| *c50673_g2* | 1548.65 | *c50673_g2_i2* | 373 | NA | no IPS match | NA | NA |
| *c45131_g1* | 1517.41 | *c45131_g1_i1* | 578 | nucleolar protein 58-like | no IPS match | NA | 79.20 |
| *c47465_g2* | 1510.38 | *c47465_g2_i1* | 684 | uncharacterized LOC101515237 | no IPS match | NA | 59.25 |
| *c39091_g2* | 1510.04 | *c39091_g2_i1* | 659 | pollen-specific protein c13-like | no IPS match | NA | 77.10 |
| *c38416_g1* | 1480.9 | *c38416_g1_i1* | 722 | NA | no IPS match | NA | NA |

FPKM- Fragments Per Kilobase of target transcript length per Million reads mapped

Mean similarity- the average similarity of the putative *C. striatus* protein and the 20 most similar proteins at NCBI

NA- not available

IPS- InterProScan
