# Supplementary material for: No evidence for Fabaceae Gametophytic self-incompatibility being determined by Rosaceae, Solanaceae, and Plantaginaceae S-RNase lineage genes
Source: BMC Plant Biol. 2015 Jun 2;15:129. doi: 10.1186/s12870-015-0497-2 (PMC4451870; doi:10.1186/s12870-015-0497-2)
Supplement: Additional file 8: — Representation of the genomic region of C. striatus CsRNase 1 (A) and CsRNase 2 (B) genes. Lines represent introns, grey boxes represent exons, and arrows indicate the most external primers used. [file 12870_2015_497_MOESM8_ESM.pdf]

Addition file 8. Expression patterns of Fabaceae *S*-lineage genes

S-lineage genes	Tissues showing expression
T2-RNase	
T. pratense Tp3	Styles with stigmas, ovaries, and leaves
T. pratense Tp6	Styles with stigmas, and leaves
C. arietinum Ca4	etiolated seedlings (XM_004486248).
M. truncatula Mt3	roots knot galls infected with *Meloidogyne hapla* (nematode)
M. truncatula Mt17	roots knot galls infected with *M. hapla*
M. truncatula Mt18	roots knot galls infected with *M. hapla*
M. truncatula Mt20	leafs and roots (Mtr.49135.1.S1_at)
C. striatus CsRNase1	ovaries, petals, pistils, leaves and fruits
C. striatus CsRNase2	ovaries, petals, pistils, leaves and fruits
C. striatus CsRNase3	ovaries, petals, leaves, and fruits
F-box genes	
C. arietinum Ca1_5	etiolated seedlings (NW_004515210)
M. truncatula Mt2_10	root knot galls infected with *M. hapla*
M. truncatula Mt2_11	leafs, petiole, stems, flowers, and roots, (*Mt2_11* - Mtr.2939.1.S1_at); immature seeds 11 to 19 days after pollination (CA990259.1)
M. truncatula Mt7_7	leafs, petiole, stems, flowers, and roots (*Mt7_7* - Mtr.14778.1.S1_at)
