# Supplementary material for: No evidence for Fabaceae Gametophytic self-incompatibility being determined by Rosaceae, Solanaceae, and Plantaginaceae S-RNase lineage genes
Source: BMC Plant Biol. 2015 Jun 2;15:129. doi: 10.1186/s12870-015-0497-2 (PMC4451870; doi:10.1186/s12870-015-0497-2)
Supplement: Additional file 9: — Correspondences between gene codes and gene names for F-box SFBB- and SFB - like genes in the vicinity of C. arietinum and M. truncatula S-RNase like genes. [file 12870_2015_497_MOESM9_ESM.pdf]

Additional file 9. Correspondences between gene codes and gene names F-box *SFBB* - and *SFB* - like genes in the vicinity of *C. arietinum* and *M. truncatula S-RNase* like genes

Gene code	Gene name
C. arietinum	
Ca1_1	*LOC101510735*
Ca1_2	*LOC101512567*
Ca1_3	*LOC101511907*
Ca1_4	*LOC101515143*
Ca1_5	*LOC101500141*
M. truncatula	
Mt2_1	*Medtr2g021050*
Mt2_2	*Medtr2g021150*
Mt2_3	*Medtr2g021160*
Mt2_4	*Medtr2g021160*
Mt2_5	*Medtr2g021170*
Mt2_6	*Medtr2g021190*
Mt2_7	*Medtr2g021210*
Mt2_8	*Medtr2g021250*
Mt2_9	*Medtr2g021250*
Mt2_10	*Medtr2g021770*
Mt2_11	*Medtr2g021800*
Mt2_12	*Medtr2g025420*
Mt7_1	*Medtr7g078840*
Mt7_2	*Medtr7g078860*
Mt7_3	*Medtr7g078900*
Mt7_4	*Medtr7g078940*
Mt7_5	*Medtr7g079160*

Mt7_6	*Medtr7g079370*
Mt7_7	*Medtr7g079640*
