# Supplementary material for: No evidence for Fabaceae Gametophytic self-incompatibility being determined by Rosaceae, Solanaceae, and Plantaginaceae S-RNase lineage genes
Source: BMC Plant Biol. 2015 Jun 2;15:129. doi: 10.1186/s12870-015-0497-2 (PMC4451870; doi:10.1186/s12870-015-0497-2)
Supplement: Additional file 10: — Primers used in this work. [file 12870_2015_497_MOESM10_ESM.pdf]

Additional file 10. Primers used in this work

Gene	Primer F	Sequence	Primer R	Sequence	T.a.º	Size (bp)
Elf1-α	Elf1-F	CCACCAACCTTGACTGGTAC	Elf1-R	CCACGCTTGAGATCCTTCAC	54	319
Tp6	5450-F	CGACTTACTTCAGTTTGC	5450-R	AAATGAGAGAAGGTTCAA	49	449 (genomic)<br>408 (cDNA)
Tp3	1821-F	TATGGTCGCAGCTCAATG	1821-R	GCTAGTAGGCTTTCAAAA	49	483 (genomic)<br>410 (cDNA)
CsRNase1	CytSRN-62F	TGCTATCTTTACAATGGC	CytRNase531R	TTACCCTTTTTATCGTCA	49	1056 (genomic)<br>487 (cDNA)
			CytSRN-379R	TTTTTCCAAGAGCAATAG		
	CytSRN-Fintrao2	AAAACACTCGTCAACAAT	CytSRN-R	ATCGCTTCCTCAACATCC	48	547
CsRNase2	CytR2-cons142F	TGTAGCAAACCATCCTCA	CytR2-445R	AGAAGCACAGACGAAGTT	52	1183 (genomic)<br>299 (cDNA)
	CytR2-824F	TGTAGCAAACCATCCTCA				
			CytR2-Rint	AGATTATTACCCCACTGC		
CsRNase3	Cy10F	ATGGGGCTTATGACTATT	Cy10R	GGAAAACGATGTGGTGAG	48	169
